# Nicotinamide N-methyltransferase inhibition mimics and boosts exercise-mediated improvements in muscle function in aged mice

**DOI:** 10.1038/s41598-024-66034-9

**Published:** 2024-07-05

**Authors:** Andrea L. Dimet-Wiley, Christine M. Latham, Camille R. Brightwell, Harshini Neelakantan, Alexander R. Keeble, Nicholas T. Thomas, Haley Noehren, Christopher S. Fry, Stanley J. Watowich

**Affiliations:** 1 Ridgeline Therapeutics, Houston, TX USA; 2 Center for Muscle Biology, College of Health Sciences, University of Kentucky, Lexington, KY USA; 3 Department of Biochemistry and Molecular Biology, University of Texas Medical Branch, Galveston, TX USA

**Keywords:** Nicotinamide N-methyltransferase, NNMT, Exercise, Proteomics, Aging, Muscle, Quality of life, Ageing, Drug development

## Abstract

Human hallmarks of sarcopenia include muscle weakness and a blunted response to exercise. Nicotinamide N-methyltransferase inhibitors (NNMTis) increase strength and promote the regenerative capacity of aged muscle, thus offering a promising treatment for sarcopenia. Since human hallmarks of sarcopenia are recapitulated in aged (24-month-old) mice, we treated mice from 22 to 24 months of age with NNMTi, intensive exercise, or a combination of both, and compared skeletal muscle adaptations, including grip strength, longitudinal running capacity, plantarflexor peak torque, fatigue, and muscle mass, fiber type, cross-sectional area, and intramyocellular lipid (IMCL) content. Exhaustive proteome and metabolome analyses were completed to identify the molecular mechanisms underlying the measured changes in skeletal muscle pathophysiology. Remarkably, NNMTi-treated aged sedentary mice showed ~ 40% greater grip strength than sedentary controls, while aged exercised mice only showed a 20% increase relative to controls. Importantly, the grip strength improvements resulting from NNMTi treatment and exercise were additive, with NNMTi-treated exercised mice developing a 60% increase in grip strength relative to sedentary controls. NNMTi treatment also promoted quantifiable improvements in IMCL content and, in combination with exercise, significantly increased gastrocnemius fiber CSA. Detailed skeletal muscle proteome and metabolome analyses revealed unique molecular mechanisms associated with NNMTi treatment and distinct molecular mechanisms and cellular processes arising from a combination of NNMTi and exercise relative to those given a single intervention. These studies suggest that NNMTi-based drugs, either alone or combined with exercise, will be beneficial in treating sarcopenia and a wide range of age-related myopathies.

## Introduction

Maintaining health, independence, and quality of life during aging largely depends on preserving muscle mass, strength, and function^1–3^. After age 50, adults lose approximately 1% of their muscle mass and strength each year, whereas, after age 60, muscle strength shows yearly declines of ~ 3%^1,2^. Severe age-linked loss of muscle mass, strength, and function, termed sarcopenia^4^, affects ~ 10% of adults aged 60 to 70^5^ and ~ 50% of adults over 80^6^. In the past decade, over 100 clinical trials have evaluated possible treatments for age-linked muscle decline and sarcopenia^7^. Attempts to increase muscle strength, mass, and function in sarcopenic adults include resistance exercise, nutritional supplements, and dietary interventions^8^. Unfortunately, although limited studies show that exercise can improve muscle strength among healthy older adults, these approaches do not appear to substantially reverse or delay sarcopenia^9–12^.

Progressive weighted wheel running (PoWeR) is a translational model of intensive concurrent (endurance and resistance) exercise that produces several skeletal muscle benefits, including increased muscle mass, improved overall muscle function, and increased muscle fiber cross-sectional area (CSA). Similar effects, though blunted, are observed when PoWeR is initiated in older (22–24-mo) animals^13–15^. We recently demonstrated that inhibiting nicotinamide N-methyltransferase (NNMT) significantly increases contractile muscle function in aged (24-month-old) mice after muscle injury^16^. Consequently, we hypothesized that NNMT inhibitor (NNMTi) treatment of aged sedentary (Sed) mice would mirror exercise-mediated muscle improvements. Moreover, if the molecular mechanisms of these two interventions are independent^16,17^, then combining NNMTi treatment with exercise would produce additive improvements in muscle function and physiology. To test these hypotheses, aged (22-month-old) mice were separated into Sed and PoWeR groups, which were further subdivided into cohorts that received once-daily dosing of saline (control) or NNMTi (5-amino-1-methylquinolinium [5A-1MQ]) for eight weeks. Measures of muscle performance (grip strength, longitudinal running distance), function (torque, fatigue), and physiology (mass, fiber type, CSA, intramyocellular lipid [IMCL] content, proteome, and metabolome) were evaluated and compared in these four cohorts.

## Results

### Grip strength and physical function measures

**Figure 1:**
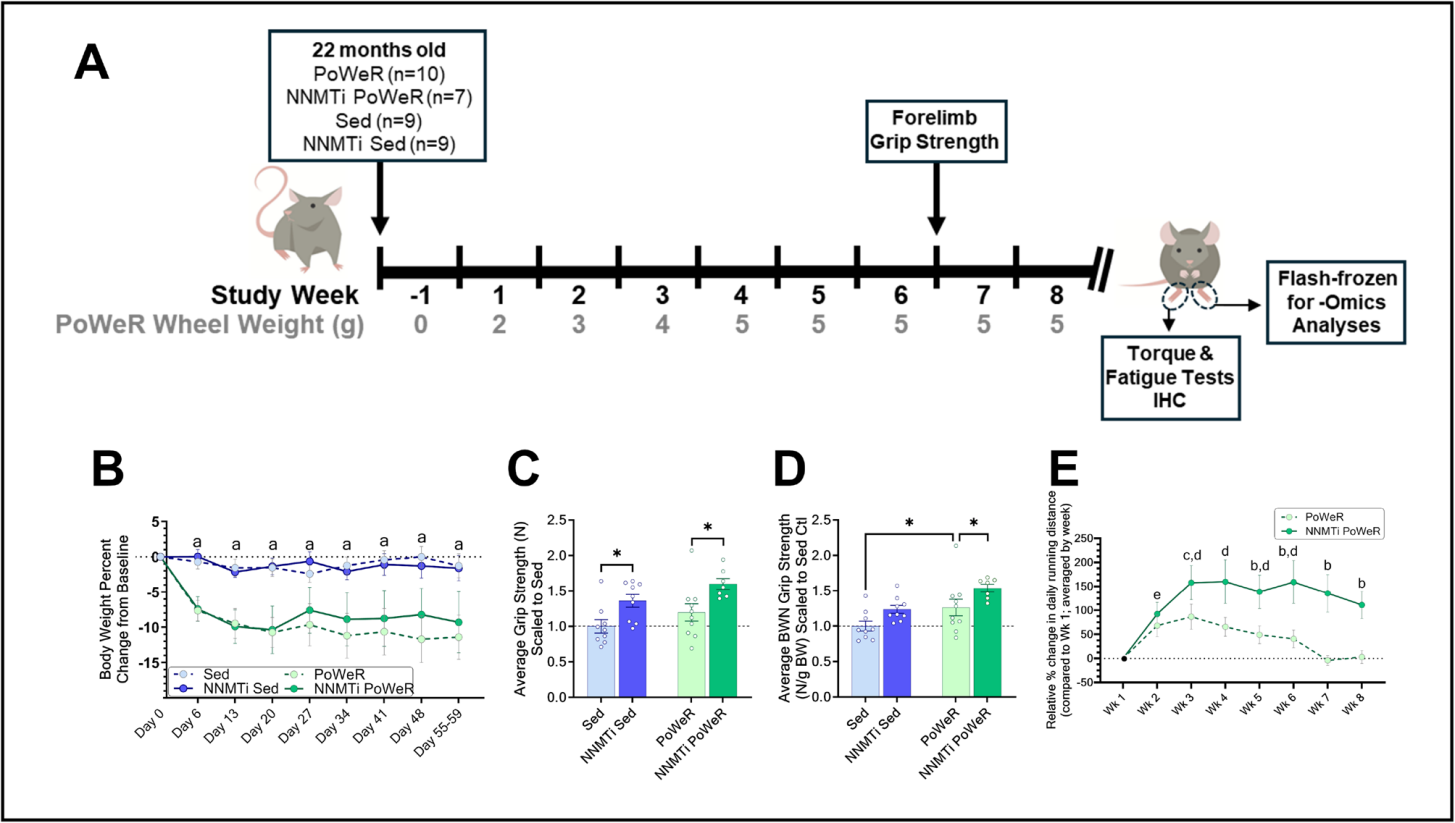
Muscle performance is enhanced by NNMTi treatment, exceeding exercise effects in Sed mice and additively improving performance in exercised mice. Twenty-two-month-old mice were randomized to PoWeR or Sed groups, with NNMTi treatment or vehicle control [**A**]. Longitudinal measures of change in body weight (BW) from baseline displayed a significant effect of study cohort (2-way repeated measures ANOVA, main effect of study cohort *P* = 0.004 [**B**]). Average grip strength, analyzed at the end of week (wk) 6, revealed that both NNMTi treatment and exercise improved grip strength (2-way ANOVA, main effect of NNMTi treatment *P* < 0.001 and exercise* P* = 0.042; [**C**]). Similar results were observed when grip strength was normalized to BW (BWN; 2-way ANOVA main effect of NNMTi treatment *P* = 0.005 and exercise *P* = 0.002; NNMTi Sed vs Sed, *P* < 0.06 [**D**]). Results for [**C**] and [**D**] are shown scaled to the Sed cohort (unscaled data shown in Supplemental Fig. 1). Relative change in average daily running distance from wk 1 in PoWeR-trained mice revealed a significant effect of group (wk 1 not included in the analysis; Mixed Effects Model: *P* = 0.0039; main effect of time not significant); posthoc testing showed significant differences between groups from weeks 5–8 [**E**]. Mean + / − SEM; * *P* < 0.05;a, PoWeR vs Sed; b, PoWeR NNMTi vs PoWeR; c, PoWeR NNMTi group, *P* < 0.05 vs. Wk 3; d, PoWeR group, *P* < 0.05 vs. Wk 7–8; e, PoWeR group, *P* < 0.05 vs. Wk 7 only.

Mice underwent the study design outlined in Fig. 1A to assess NNMTi treatment and exercise interventions for age-associated muscle weakness. Both PoWeR cohorts had an ~ 8% decline in body weight (BW) in study week 1, while the Sed groups’ BWs remained essentially unchanged throughout the study (Fig. 1B, Supplemental Fig. 1).

Forelimb grip strength was chosen to measure muscle function since it has been repeatedly shown that C57BL/6 mouse forelimb grip strength begins to decline at or before 24 months of age^18–20^. Additionally, progressive weighted wheel running has been shown to significantly improve forelimb grip strength in young (< 8-month-old) C57BL/6 mice^21^. Grip strength measured during week 6 of our study showed main effects of both exercise and NNMTi treatment. Notably, the NNMTi-treated Sed and PoWeR cohorts had ~ 40% and 20% greater grip strengths, respectively, relative to the untreated (control) Sed cohort. Moreover, the NNMTi-treated PoWeR cohort had ~ 60% greater grip strength than the Sed cohort (Fig. 1C, Supplemental Fig. 1), suggesting that combining NNMTi and PoWeR treatments produced additive effects. Normalizing grip strength to BW did not dramatically change the observed improvements noted in the NNMTi-treated cohorts relative to the untreated cohorts. However, BW normalization did increase the relative strength in the untreated PoWeR cohort since these animals had the lowest BWs relative to the other cohorts (Fig. 1D, Supplemental Fig. 1).

During the first three weeks of our study, both PoWeR cohorts showed increases in their average weekly running distances (Supplemental Fig. 1). Given differences in the initial average weekly running distance between the cohorts, data were analyzed as the change in running relative to week 1 (Fig. 1E, Supplemental Fig. 1). By week 3, NNMTi-treated PoWeR mice were running an additional 3.0 km/day compared to week 1 (a 250% increase), whereas the PoWeR cohort only ran an extra 2.3 km/day compared to week 1 (a 170% increase). The NNMTi-treated PoWeR mice largely maintained their improved running capacity through week 8, running ~ 1.8 km/day more in week 8 compared to week 1 (Supplemental Fig. 1). In contrast, by weeks 7 and 8, the average daily running distance of the PoWeR cohort had returned to week 1 levels (Fig. 1E). Importantly, the change in running distance relative to week 1, averaged by week, throughout the study showed a significant difference between treatment group, with the NNMTi-treated PoWeR group running significantly more than the PoWeR group weeks 6–8 (Fig. 1E). These results suggest that NNMTi treatment improved tolerance to, or recovery from, intensive exercise.

Peak plantarflexor torque and BW-normalized (BWN) peak plantarflexor torque (measured at the end of week 8) were not significantly changed by NNMTi or PoWeR (Supplemental Fig. 1). Fatigue, measured as the number of contractions ≥ 70% or ≥ 50% of the maximum peak torque observed during a 54-bout test, and cumulative work across the fatigue test, were used to further assess plantarflexor complex (i.e., gastrocnemius, plantaris, and soleus) function. A clear trend emerged for reduced susceptibility to fatigue in NNMTi-treated mice relative to matched controls (Supplemental Fig. 1; Supplemental Table 1; main effect of NNMTi treatment on repetitions above 70% in the fatigue test, *P* = 0.098). This may directly relate to the significantly reduced cumulative work in NNMTi-treated mice during the fatigue task since one explanation for the reduced cumulative work is lower peak torques during each contraction (Supplemental Fig. 1).

### Muscle weights and composition

Plantarflexor complex muscle masses were BWN to account for terminal BW differences across cohorts (Table 1; Supplemental Table 1). The gastrocnemius accounted for ~ 83% of the plantarflexor complex mass. BWN gastrocnemius masses were > 20% larger in the PoWeR cohorts compared to matched Sed cohorts and were unaffected by NNMTi treatment (Supplemental Fig. 2). Similarly, BWN plantaris and soleus masses were, on average, ~ 37% greater in the PoWeR cohorts than in matched Sed cohorts; there were no statistically significant effects (Supplemental Fig. 2). Additionally, BWN heart masses were significantly increased in the PoWeR cohorts compared to the Sed cohorts and were unaffected by NNMTi treatment relative to controls (Supplemental Fig. 2), suggesting exercise-induced cardiac hypertrophy.

**Table 1:** Summary of muscle mass, fiber CSA, and fiber type results.

	Sed cohort	NNMTi-treated sed cohort	PoWeR cohort	NNMTi-treated PoWeR cohort
Gastrocnemius mass (body weight normalized)^a^	2.8 ± 0.5	3.0 ± 0.6	3.8 ± 0.3	3.6 ± 0.2
Gastrocnemius mass (raw)^a,b^	75.5 ± 11.5	89.2 ± 13.8	97.2 ± 8.8	103.5 ± 7.8
Gastrocnemius CSA (pooled)^c^	1481 ± 287.8	1462 ± 200.3	1513 ± 204.2	1892 ± 138.7
Gastrocnemius CSA Type IIb^c^	1625 ± 273.2	1617 ± 222.1	1690 ± 192.2	2128 ± 167.3

Significant (adjusted *p* < 0.05) comparisons are indicated as follows: ^a^PoWeR cohort vs. Sed cohort; ^b^NNMTi Sed cohort vs. Sed cohort; ^c^NNMTi PoWeR cohort vs. PoWeR cohort.

In aged (24-month-old) mice, PoWeR has been reported to transition muscle from fast-twitch (Type II) to slow-twitch (Type I) muscle fibers^13^. The CSAs of these fiber types have been significantly correlated with leg extension strength but not leg press strength, and whole-quadriceps muscle CSA in older humans^22^. We completed detailed analyses of muscle fiber type and CSA within the plantarflexor complex (representative histology, Supplemental Fig. 2) to determine if these measures responded to NNMTi treatment or exercise. There were no statistically significant changes in the relative abundance of each fiber type in the gastrocnemius muscle (Supplemental Fig. 2). The CSA of pooled gastrocnemius fiber types was significantly greater (i.e., ≥ 25%) in the NNMTi-treated PoWeR cohort compared to all other cohorts (Supplemental Fig. 2). This pronounced effect in a large muscle led us to focus on the gastrocnemius for downstream analyses. The increased CSA of pooled fibers from the NNMTi-PoWeR-treated cohort resulted predominantly from increased Type IIb fiber CSA since these fibers account for ~ 72% of gastrocnemius fibers. Additionally, Type I, IIa, IIb, IIa/b, and IIx fiber CSAs from the NNMTi-PoWeR-treated cohort were increased on average compared to the other cohorts (Supplemental Fig. 2). NNMTi or exercise treatment alone did not change the CSA of gastrocnemius fiber types.

In the plantaris, the PoWeR cohort had ~ 14% fewer Type IIb fibers but ~ 9% more Type IIa/b fibers relative to all other cohorts (Supplemental Fig. 2). Additionally, the CSAs of pooled plantaris muscle fibers were ~ 21% larger in the PoWeR cohorts than the Sed cohorts (Supplemental Fig. 2). Exercise-mediated increases in pooled plantaris fiber CSA arose from statistically significant increases in Type IIa, Type IIb, and Type IIx fiber CSAs (Supplemental Fig. 2); these same fiber types showed statistically significant CSA changes in the gastrocnemius and the increased Type IIa CSA aligns with previous work^13^.

In the soleus, the only significant fiber type-switching observed was a near-doubling of Type I/IIa fiber percentage, balanced by a non-significant reduction in Type IIa fibers in the NNMTi-treated PoWeR cohort relative to other cohorts (Supplemental Fig. 2). This limited fiber type-switching differed from previous work^23,24^ but may relate to differences in mouse ages or PoWeR experiment design. In contrast to the other muscles examined, a sizeable fraction of soleus fibers were Type I, with Type IIb fibers absent altogether (Supplemental Fig. 2). The CSA of the pooled soleus muscle fibers was significantly greater in the PoWeR cohorts compared to the Sed cohorts (Supplemental Fig. 2), supported by increases of ~ 30% and ~ 32% in Type I and Type IIa fiber CSAs, respectively, in exercised cohorts compared to Sed cohorts (Supplemental Fig. 2). The exercise-mediated increases in Type IIa and Type I CSA are consistent with previous work^13^. For Type IIx fiber CSA, the soleus mimicked the gastrocnemius with a significant main effect of exercise, but no significant differences emerged in pairwise comparisons (Supplemental Fig. 2); there were no statistically significant changes to Type I/IIa CSA despite trends for increased CSA with NNMTi treatment as well as exercise (Supplemental Fig. 2).

**Figure 2:**
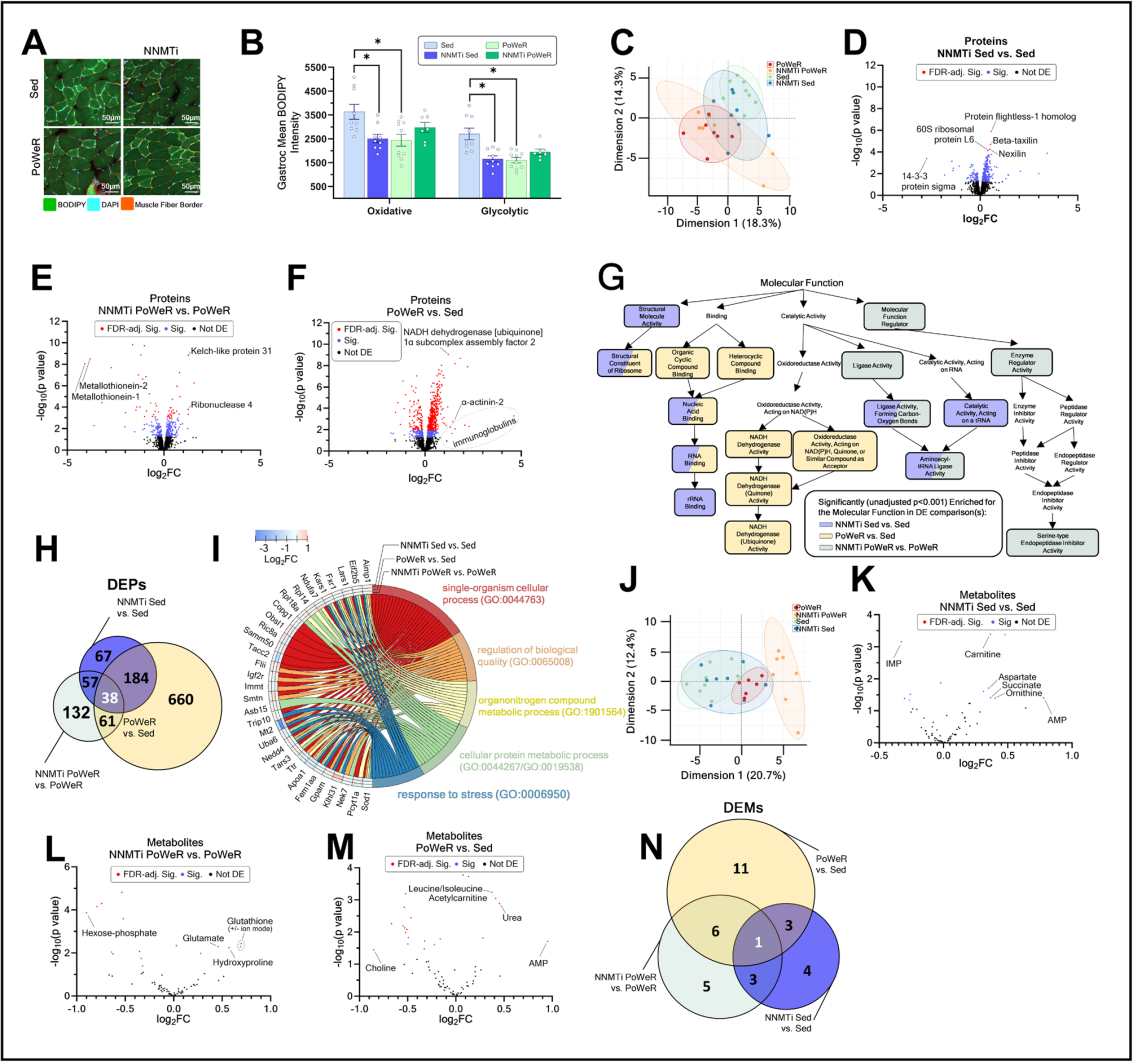
Gastrocnemius histology, proteomics, and metabolomics suggest that NNMTi treatment in Sed mice recapitulates many exercise effects, while NNMTi treatment in exercising mice has disparate effects. Gastrocnemius (gastroc) histology of intramyocellular lipid (IMCL) content (representative histology, [**A**]) revealed that oxidative fibers demonstrate a significant NNMTi x exercise (PoWeR) interaction (2-way ANOVA main effect of interaction:* P* = 0.002), with significantly greater IMCL content in oxidative fibers of the Sed cohort relative to the NNMTi-treated Sed cohort or PoWeR cohort [**B**]. Gastroc glycolytic fibers demonstrate a stronger effect, with significant main effects of NNMTi treatment, PoWeR, and an interaction (2-way ANOVA:* P* = 0.039, *P* = 0.019, and *P* < 0.001, respectively); on average, there was greater IMCL content in the Sed cohort than all others [**C**]. Principal component analysis (PCA) of the gastroc proteome revealed distinct protein expression in the Sed cohort and PoWeR cohort, with the NNMTi-treated Sed cohort substantially overlapping across these cohorts and the NNMTi-treated PoWeR cohort largely overlapping the PoWeR cohort [**D**]. The differentially expressed proteins (DEPs) revealed that exercise most dramatically changed the proteome. Still, several NNMTi-regulated proteins with large fold changes are relevant to muscle physiology [**E**–**G**]. GOrilla analyses exploring the molecular functions enriched (GOrilla-computed FDR-adjusted *P* < 0.05) for the DEPs supported that many NNMTi-mediated effects in Sed mice impinge upon the same functions as exercise [**H**]. The 38 DEPs common to all three comparisons are associated with metabolic processes and stress response [**I**, **J**]. The metabolomic PCA mostly recapitulates the proteomic PCA but shows a unique separation of the NNMTi-treated PoWeR cohort from the PoWeR cohort [**K**]. Similarly, metabolomic results suggested that exercise changes the expression of the greatest number of metabolites but that many NNMTi-regulated metabolites with the greatest fold change are relevant to muscle physiology and metabolism [**L**–**N**]. Only one differentially expressed metabolite (DEM) was common in all comparisons [**O**]. Mean + / − SEM; *P and FDR-adjusted *P* < 0.05; *rRNA* ribosomal RNA; *tRNA* transfer RNA.

In humans, IMCL content has been reported to decrease during prolonged submaximal exercise^25,26^, increase in highly trained states^25,27–31^, and inversely correlate with insulin sensitivity in sedentary adults^27^. Herein, we evaluated gastrocnemius IMCL content using BODIPY (Fig. 2A). IMCL was greatest in the Sed cohort, irrespective of whether measurements were in glycolytic (Type IIb, Type IIx) or oxidative (Type I, Type IIa) fibers^32,33^. Importantly, IMCL levels in glycolytic and oxidative muscle fibers of NNMTi-treated Sed mice were reduced by > 30% compared to the Sed cohort, reaching levels similar to those observed in the PoWeR cohorts (Fig. 2B). Thus, NNMTi-treatment of aged Sed mice likely modulates lipid synthesis and metabolism (*e.g.*, fatty acid oxidation, as supported by the upregulation of the protein carnitine essential for mitochondrial fatty acid uptake^34^) in a manner similar to exercise.

### Proteomic measures in aged muscle

Global proteomic analysis of the gastrocnemius was completed to uncover molecular mechanisms responsible for the observed differences in muscle function, mass, and composition. A common set of 2,512 proteins were identified, quantified, and analyzed in all samples. Principal component analysis (PCA) of the quantified proteins showed distinct Sed cohort and PoWeR cohort clusters. The NNMTi-treated Sed cohort substantially overlapped across the Sed cluster and PoWeR cluster. The NNMTi-treated PoWeR cluster largely overlapped the PoWeR cluster (Fig. 2C).

We identified 346 differentially expressed proteins (DEPs; 281 upregulated, 65 downregulated; Fig. 2D) from NNMTi treatment in the Sed context (NNMTi-treated Sed vs. Sed), 288 DEPs (182 upregulated, 106 downregulated; Fig. 2E) from NNMTi treatment in the exercise context (NNMTi-treated PoWeR vs. PoWeR), and 943 DEPs (734 upregulated, 209 downregulated; Fig. 2F) from exercise (PoWeR vs. Sed). The number of DEPs between study cohorts aligned with the PCA plot; the comparisons with the largest number of DEPs (PoWeR vs. Sed) had the least PCA plot overlap, while those with fewer DEPs had greater overlap.

Several DEPs in the NNMTi-treated Sed cohort vs. the Sed cohort were associated with 60S and 40S ribosomal complexes and were significantly upregulated (Supplemental Table 2). Other upregulated DEPs across these cohorts were involved in cytoskeletal structure and regulation (*e.g.*, protein flightless-1 homolog, nexilin, coronin-1A, leiomodin-3, obscurin-like protein 1), immune signaling (*e.g.*, immunoglobulin [Ig] heavy variable 14–4, Ig kappa variable 4–63, Ig lambda constant 1), and ubiquitination (*e.g.*, tripartite motif-containing protein 16, ubiquitin-conjugating enzyme E2 R2, Ubiquitin carboxyl-terminal hydrolase 13). The most upregulated DEPs in NNMTi-treated Sed vs. Sed mice included β-taxilin, desmin, and the leukemia inhibitory factor receptor. The most downregulated DEPs included serine protease inhibitors (*i.e.*, murinoglobulin-1 [Mug1], serine protease inhibitor [serpin] A3M) and metallothionein-1 and -2 (MT1, MT2; Supplemental Table 2).

The most highly upregulated DEPs between NNMTi-treated PoWeR and PoWeR mice included ubiquitin carboxyl-terminal hydrolase 13, β-taxilin, Kelch-like protein 31, smoothelin, nexilin, and protein tyrosine phosphatase type IVA 2. The most downregulated DEPs included MT1 and MT2, peptidyl-prolyl cis–trans isomerase FKBP5, transforming acidic coiled-coil-containing protein 2, and Mug1 (Supplemental Table 2). The serpin A1e and A3n proteins were significantly downregulated by NNMTi treatment only in the exercise context, indicating that NNMTi treatment may impact additional unique pathways when combined with exercise. These DEPs and their associated pathways could be responsible for increasing gastrocnemius Type IIb fiber CSA, as was only observed in NNMTi-treated PoWeR mice. They may offer new drug targets for increasing muscle mass and function.

NNMTi treatment in the Sed or the exercise context significantly changed the expression levels of only a limited number of proteins (on average, treatment modulated ~ 10% of the > 2,500 proteins analyzed; Supplemental Table 2), although many of these DEPs were common to both comparisons. Three proteins, BCL2 interacting protein 3 (Bnip3), MT1, and MT2, significantly downregulated by NNMTi treatment in both the Sed and exercise contexts, have been definitively linked to skeletal muscle atrophy^35–39^. Furthermore, each protein’s expression level was significantly and negatively correlated with the gastrocnemius pooled CSA (Spearman’s correlation, *P* < 0.05 for each, simple linear regression R^2^ = 0.331, 0.423, and 0.462 for Bnip3, MT1, and MT2, respectively).

Exercise treatment alone (PoWeR vs. Sed) resulted in ~ 38% of all analyzed proteins being differentially expressed (Supplemental Table 2). Some mitochondrial proteins (*e.g.*, mitochondrial chaperone BCS1, mitochondrial nicotinamide adenine dinucleotide [NAD] transporter SLC25A51), including several involved in β-oxidation (*e.g.*, acyl-CoA dehydrogenases), were uniquely upregulated by exercise alone. However, many mitochondrial proteins (*e.g.*, hydrogenated NAD [NADH] dehydrogenase subunits, 39S ribosomal proteins) and several immunoglobulins (*e.g.*, kappa variable 4–63, lambda constant 1, heavy variable 14–4) were consistently upregulated by exercise and NNMTi treatment of Sed mice.

DEPs were mapped to GO-defined molecular functions to elucidate the cellular processes that respond to NNMTi treatment, exercise, or their combination (Fig. 2G). NNMTi treatment of Sed mice was significantly associated with nucleic acid and ribosomal RNA binding, and ribosome structure. Interestingly, the ribosome structure and nucleic acid binding molecular functions were also enriched for exercise-produced DEPs (PoWeR vs. Sed). NADH dehydrogenase activity was independently enriched for exercise-produced DEPs. Many functions enriched for DEPs from NNMTi treatment in the exercise context were also enriched for DEPs from NNMTi treatment in the Sed context, but serine-type endopeptidase inhibitor activity (*e.g*., serpin) was uniquely enriched for DEPs from NNMTi treatment in the PoWeR context.

Approximately two-thirds of the DEPs observed in the NNMTi-treated Sed cohort relative to the Sed cohort overlapped with DEPs from the PoWeR cohort relative to the Sed cohort (Fig. 2H); all but two DEPs had the same directional change in expression. In contrast, fewer than 25% of DEPs associated with exercise (PoWeR vs. Sed) overlapped proteins modulated by NNMTi treatment of Sed mice. Furthermore, most DEPs resulting from NNMTi treatment in the exercise context did not coincide with DEPs associated with exercise alone; for those that did, ~ 60% had expression level changes in opposite directions. This suggests that many DEPs altered by exercise alone may affect cellular functions unrelated to muscle growth.

The 38 DEPs common to all three comparisons (Fig. 2H) were mapped to GO terms to determine potential molecular functions common to all interventions. Most DEPs common across all treatments were associated with stress response or metabolism (Fig. 2I).

### Metabolomic measures in aged muscle

Global metabolomic analysis of the gastrocnemius identified and quantified 69 metabolites common to all samples. PCA of the metabolites produced distinct clusters for the Sed cohort and PoWeR cohort (Fig. 2J), similar to clustering produced by proteomic PCA. This suggests a strong influence of exercise on muscle metabolism. Additionally, the NNMTi-treated Sed cohort cluster enveloped the Sed cohort and PoWeR cohort clusters, suggesting that NNMTi treatment might shift the metabolome of a Sed mouse closer to that of a PoWeR mouse, perhaps through increased adenosine monophosphate (AMP) and decreased lactate, inosine monophosphate, and glycerophosphocholine. Unlike the proteome, the PCA cluster of the NNMTi-treated PoWeR metabolome was well-separated from all others, driven by unique downregulation of methionine, proline, serine, asparagine, hexose-phosphate, and D-glyceraldehyde-3-phosphate, and suggesting that additive effects of NNMTi treatment and exercise significantly influence muscle metabolism.

Differentially expressed metabolites (DEMs) were determined similarly to DEPs. Volcano plots highlight 11 DEMs from NNMTi treatment in the Sed context (NNMTi-treated Sed vs. Sed; 6 upregulated, 5 downregulated; Fig. 2K), 15 DEMs from NNMTi treatment in the exercise context (NNMTi-treated PoWeR vs. PoWeR; 5 upregulated, 10 downregulated; Fig. 2L), and 21 DEMs from exercise (PoWeR vs. Sed; 6 upregulated, 15 downregulated; Fig. 2M). The overlap of these metabolites (Fig. 2N) showed 4 metabolites common across NNMTi treatment contexts, with carnitine upregulated and glycerophosphocholine and glutamine downregulated. Creatine was upregulated in the PoWeR context but downregulated in the Sed context.

## Discussion

Resistance or combined resistance and endurance exercise are the current standard of care treatments that attempt to slow age-associated loss of muscle mass and strength^40,41^. This study showed that NNMTi treatment could surpass the muscle health benefits of resistance and endurance exercise. NNMTi treatment dramatically increased grip strength in both aged Sed and aged exercised animals, suggesting that treatment prevents age-associated loss of strength and actively rebuilds strength. Importantly, NNMTi treatment almost doubled the increase in muscle strength compared to exercise alone. Moreover, the increases in muscle strength resulting from NNMTi treatment or exercise were additive, with the strength of the NNMTi-treated exercise cohort being increased by ~ 60% compared to the Sed cohort.

Grip strength has been associated with glutathione homeostasis^42^, and glutathione regulation appears to be modulated, at least in the NNMTi-treated exercise group relative to the exercise group. Although both grip strength and plantarflexor strength heavily rely on force exertion^43^, the prioritization of the rate of force generation may differ for the muscle groups. Thus, unsurprisingly, peak torque did not correlate with grip strength in any cohort (data not shown). Prior work demonstrates progressive declines in many measures of functional capacity and strength with increasing age, although it is noted that the declines vary substantially and in a non-linear manner^18,44^. In response to voluntary wheel running (both weighted and unweighted) in aged mice, improvements in grip strength without concomitant improvements in plantarflexor strength have been observed, similar to our findings^45–47^. Thus, incongruency between these strength measures may be expected.

NNMTi-treated exercised mice increased their initial running capacity and sustained this increase throughout the study. In contrast, control-treated exercised mice showed a temporary improvement in running capacity that was not sustained throughout the study. This suggests that NNMTi treatment enhanced tolerance to exercise, consistent with the tendency for reduced susceptibility to muscle fatigue observed in NNMTi-treated animals. The sustained running capacity in NNMTi-treated animals may also be tied, in part, to improved recovery from exercise-associated injury, consistent with work in a different muscle injury model^16^.

**Figure 3:**
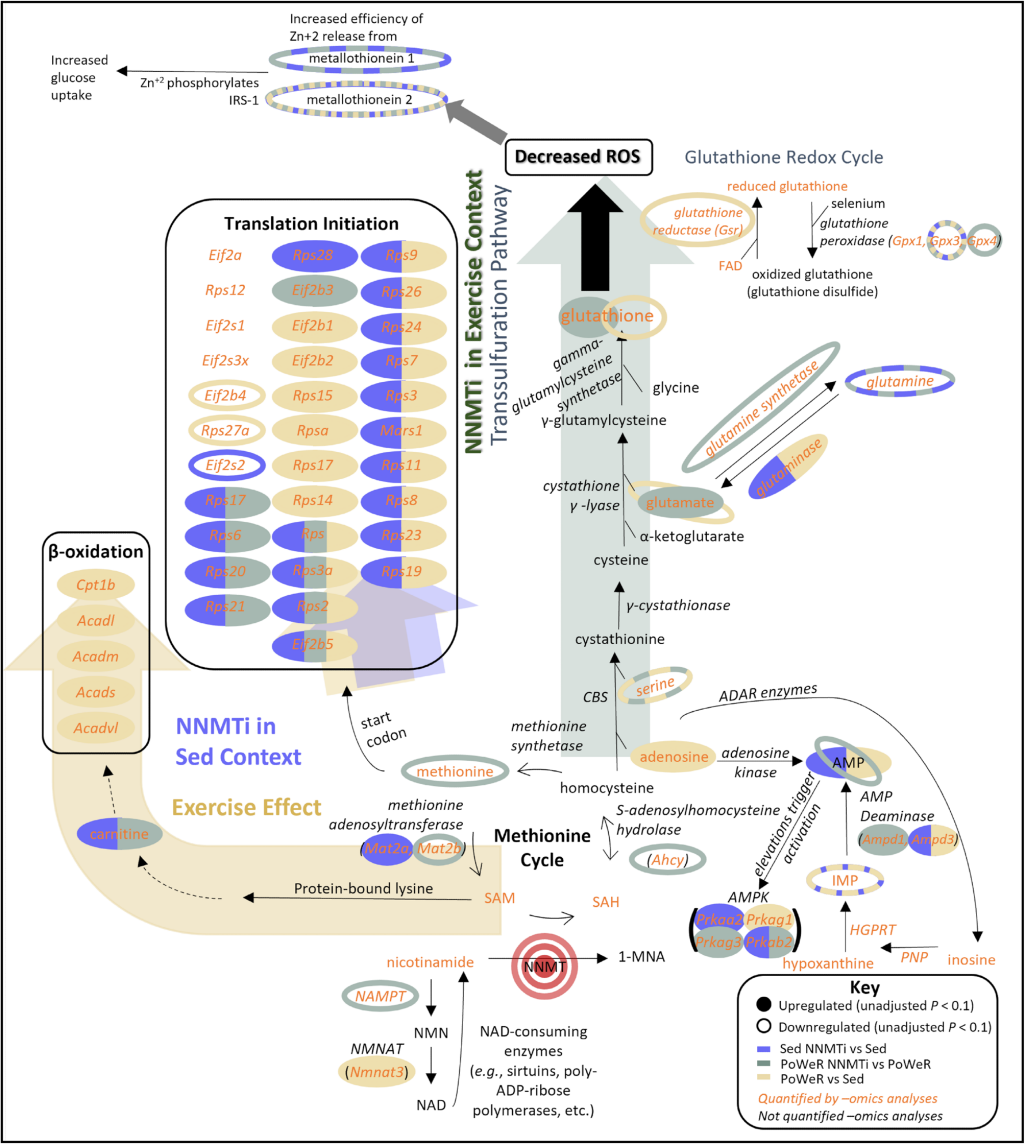
–Omics results mapped to potential downstream pathways suggest different NNMTi treatment-mediated mechanisms in exercised and Sed contexts. In Sed mice, NNMTi treatment upregulates several components critical to protein translation. In exercised (PoWeR) mice, NNMTi treatment upregulates transsulfuration pathway components, suggesting increased protection against reactive oxygen species damage. Critical elements of protein translation and β-oxidation are upregulated in exercised mice relative to Sed mice. Together, the data support that downstream mechanisms of NNMTi treatment vary with the cellular context and that some mimic exercise. Gene names corresponding to the proteins are italicized when listed. Other abbreviations are: *1-MNA* 1-methylnicotinamide; *ADAR* adenosine deaminase acting on RNA; *CBS* cystathionine β-synthase; *HGPRT* hypoxanthine–guanine phosphoribosyltransferase; *IMP* inosine monophosphate; *IRS-1* insulin receptor substrate 1; *NAMPT* nicotinamide phosphoribosyltransferase; *NMN* nicotinamide mononucleotide; *NMNAT* NMN adenylyltransferase; *PNP* purine nucleoside phosphorylase; *SAH* S-adenosyl-L-homocysteine; *SAM* S-adenosylmethionine.

NNMTi treatment and intensive exercise were associated with numerous upregulated proteins involved in protein translation (Fig. 3, Supplemental Fig. 3). This likely accounts for the increased gastrocnemius weight observed with exercise and the increased CSA observed with NNMTi treatment. Protein upregulation may relate to the fact that AMP was upregulated in the exercised and NNMTi-treated Sed cohorts, each relative to the Sed cohort. AMP’s upregulation could drive AMPK activation, which has been implicated in energy-sensing, muscle hypertrophy, and protein translation^48–50^. Additionally, high IMCL content correlates with poorer muscle quality/functional performance^51^, increased skeletal muscle insulin resistance^52^, and reduced myofiber contraction velocity, force, and power^53^. NNMTi treatment and intensive exercise independently reduced IMCL content and increased grip strength relative to the Sed cohort, suggesting improved muscle quality with both interventions.

There were significant increases in plantarflexor complex muscle masses and CSA in the exercised cohorts, with the CSA increases occurring mainly in glycolytic (Type IIb, Type IIx) fibers of the gastrocnemius and plantaris and oxidative (Type I, Type IIa) soleus fibers (Table 1). These results align with previous work in aged mice^13^, which showed an exercise-mediated increase in the BWN soleus weight and soleus pooled CSA and a trend (*P* = 0.08) for an increase in plantaris pooled CSA in conjunction with no change to gastrocnemius pooled CSA^13^. Surprisingly, NNMTi-treated Sed mice did not have significantly increased BWN plantarflexor masses or fiber CSAs relative to the Sed cohort. However, NNMTi-treated PoWeR mice had greatly increased gastrocnemius Type IIb fiber CSA relative to all other cohorts, partially explained by our proteomics results.

NNMTi treatment in both Sed and exercise contexts modulated muscle function-associated molecular mechanisms and cellular processes distinct from and common to those modulated by exercise alone (Supplemental Fig. 3). Among the dominant cellular processes that were modulated in the NNMTi treatment groups were protein translation and ribosomal biogenesis (Fig. 3, Supplemental Fig. 3); this is intriguing given observations of age-associated dysregulation of protein homeostasis synthesis^54,55^ and may relate to age-associated changes such as muscle denervation or changes to a variety of environmental signals known to influence mTORC1^56^. Additionally, a common set of DEPs were observed following NNMTi treatment of Sed and PoWeR animals, including upregulated desmin, a muscle-specific structural component that may play a role in organelle positioning^57^, and downregulated Bnip3, whose expression is highly linked to muscle atrophy, age-associated inflammation, and mTOR regulation^58–60^. NNMTi treatment was also associated with downregulated Mug1, which has been reported to be upregulated after muscle injury^61^.

Importantly, several DEPs were uniquely associated with NNMTi treatment in the Sed context as opposed to the PoWeR context; among these DEPs were upregulated proteins required to initiate translation, including 40S subunits^62^ and the leukemia inhibitory factor receptor that regulates inflammation during skeletal muscle regeneration^63^. DEPs associated with NNMTi-treated Sed mice mapped to molecular functions related to ribosomal RNA biogenesis and binding, aminoacyl-transfer RNA ligase activity, and ribosome structural constituents. Although the analyzed proteome was specific to the gastrocnemius, this highly plastic muscle may develop greater aerobic respiration capacities to increase ATP production, as supported by the decreased lactate in NNMTi-treated Sed mice. Future studies will probe the dynamic reprogramming of cellular respiratory capacities of aerobic and anaerobic muscles in response to NNMT inhibition.

Only 15% of DEPs in NNMTi-treated exercised mice were also exercise-regulated DEPs with expression modulated in the same direction. This suggests that NNMTi treatment of exercised mice may modulate cellular processes that are not highly affected by exercise (*e.g.*, response to hormone [Supplemental Fig. 3]) and cellular processes that respond to exercise (*e.g.*, the transsulfuration pathway, Fig. 3). Pyruvate dehydrogenase (acetyl-transferring) kinase isozyme 4, serpin A3N, muscle RING-finger protein-1 (MuRF1, also called E3 ubiquitin-protein ligase Trim63), and glutamine synthetase were uniquely differentially expressed only in NNMTi-treated exercised mice, suggesting these proteins may require an NNMTi-exercise interaction. Serpin A3N has recently been proposed as a plasma biomarker for sarcopenia and muscle atrophy^64^, and MuRF1 is thought to promote the degradation of myofibrillar proteins^58^. The fact that MuRF1 was downregulated only in the NNMTi-treated PoWeR cohort nicely aligns with this cohort being the only one with significantly increased gastrocnemius fiber CSA. The downregulation of glutamine synthetase in this cohort may underlie the upregulated glutamate and glutathione observed, suggesting that NNMTi treatment combined with exercise upregulates components of the methionine transsulfuration pathway (Fig. 3). Exercise^65^ and older age^66^ typically reduce glutathione abundance in skeletal muscle. Correspondingly, the PoWeR cohort had lower glutathione levels than their Sed counterparts. Glutathione supplementation has been shown to improve aerobic metabolism^67^. Thus, the upregulation of glutathione and downregulation of AMP in the NNMTi-treated PoWeR cohort suggests a likely improvement in aerobic metabolism and a potential mechanistic rationale for the sustained running capacity observed in this cohort.

Intensive exercise was associated with significant changes in the expression levels of ~ 40% of the 2,512 proteins analyzed, underscoring the ability of exercise to modulate numerous and diverse cellular processes. Given the large number of DEPs associated with exercise, it was unsurprising that ~ 65% of NNMTi-treated Sed mice vs. Sed mice DEPs were also differentially expressed by exercise. The majority (> 78%) of DEPs common to NNMTi treatment and exercise were regulated in the same direction, suggesting that many cellular responses were similar between the two interventions. However, it is notable that exercise was linked to the expression of several acyl-CoA dehydrogenases that were not changed with NNMTi treatment, suggesting exercised skeletal muscles shift toward β-oxidation as a fuel source.

Surprisingly, only a minimal number of DEPs were common among the three comparisons (NNMTi-treated Sed vs. Sed, NNMTi-treated PoWeR vs. PoWeR, PoWeR vs. Sed), with most of these DEPs showing the same regulation across all comparisons. The DEPs common across these comparisons were associated with mitochondrial functioning (e.g., upregulated mitochondrial glycerol-3-phosphate acyltransferase 1), protein translation (e.g., upregulated 60 s ribosomal proteins L14, L18a, L7a; upregulated lysine and leucine transfer RNA ligases), proteasomal degradation (e.g., upregulated E3 ubiquitin-protein ligase NEDD4), detoxification of reactive oxygen species (e.g., downregulated superoxide dismutase), and motor nerve regeneration (e.g., upregulated β-taxilin). Additionally, all treatments that increased grip strength showed significantly decreased expression of cysteine-rich metallothioneins (MTs) and increased expression of Kelch-like protein 31, the latter linked to skeletal myogenesis^68^. MT2 was strongly downregulated (log_2_FC <  − 1) in all three comparisons, whereas only NNMTi treatment strongly downregulated MT1 expression. MTs have been suggested to regulate cellular zinc concentration and protect cells from inflammation by modulating oxidative stress^69^. Moreover, MT downregulation protects against glucocorticoid-induced muscle atrophy^70^, increases Type IIb fiber hypertrophy and muscle torque^70^, and impacts mitochondrial function by regulating metal ions^71^. Although the exact mechanism linking NNMT inhibition or exercise to MT downregulation is unknown, it is believed that MT1 and MT2 downregulation activates the Akt/mTOR pathway^70^, which drives skeletal muscle hypertrophy and reduced muscle atrophy^72^. Atrophy-associated proteins MT1, Serpin A3N, MuRF1, and Bnip3 were unmodified by exercise alone. However, they were significantly downregulated by NNMTi treatment combined with exercise, suggesting that the knockdown of these proteins, perhaps in combination with MT2, may synergistically prevent atrophy or drive the increased muscle fiber CSA observed in the gastrocnemius muscle.

Extending these studies to male mice, other species, younger animals, and studies with longer durations of treatment and stopping treatment but continuing to monitor the mice can further establish the larger impact of NNMTi treatment on improving muscle strength and function during aging. Studies adding other compounds modifying NNMT-associated pathways, such as nicotinamide or nicotinamide riboside, as a comparator will further differentiate NNMTi mechanisms^73^. An increase in NAD is one potential downstream mechanism for the muscle benefits of NNMTi treatment. Within the NAD salvage pathway, NNMT inhibition prevents nicotinamide depletion, allowing it to be converted to NAD. Careful quantification of NAD salvage metabolites and modulation through conditional knockout animals may help elucidate NAD’s direct role in NNMTi-mediated effects. To help determine the pleiotropic effects of NNMTi on protein expression and cellular processes, future studies will focus on proteomic and metabolomic analyses of different muscle groups and cells collected from rodents of varied ages, sexes, and species, similar to studies unraveling the role of specific proteins (e.g., mTOR1^56^)on skeletal muscle mass regulation. These will enable an integrated understanding of age-linked muscle remodeling and advance NNMTi drugs as novel treatments to greatly improve and sustain muscle function in older adults.

## Methods

### Chemicals

The NNMTi 5A-1MQ was synthesized as reported previously^74^. The NNMTi was diluted in 0.9% NaCl sterile saline solution to create a stock solution of 2 mg/mL. Fresh solutions were made weekly. Reagent details are in Supplemental Table 4.

### In vivo experiments

Animal procedures were performed per all national and local guidelines and regulations, the Guide for the Care and Use of Laboratory Animals, the ARRIVE guidelines, and the approval of the Institutional Animal Care and Use Committee (protocol #2019-3301) at the University of Kentucky.

Aged (22-mo) female mice (n = 35), obtained from the US NIH National Institute on Aging (NIA) Aged Rodent Colony, were randomly assigned to one of four groups: Sed (saline; n = 9), Sed NNMTi-treated (10 mg/kg BW; n = 9), progressive weighted wheel running (PoWeR; saline; n = 10), and PoWeR NNMTi-treated (10 mg/kg BW; n = 7). Our published^16^ and unpublished studies show this NNMTi dose promotes effective muscle regeneration post-injury in aged mice; Supplemental Fig. 4 shows data from a pharmacokinetic study for NNMTi 5A-1MQ; additional method details are included in Supplemental File 1. Results suggest rapid and substantial absorption of 5A-1MQ post-subcutaneous dosing, a robust half-life (~ 7 h), and an absence of accumulation with repeat dosing.

Female mice were chosen for this study since previous work have shown that they run longer distances than males in this age range^75^, including when the wheel is weighted in the PoWeR model^76^. Since mice were randomized by cage, slight differences across cohorts in BW mean and variation existed at the study start, with the mice assigned to NNMTi treatment roughly 10% heavier than their control counterparts. All mice received daily subcutaneous injections of saline or NNMTi and were weighed weekly to adjust dose volumes.

Group-housed Sed cohorts were compared to singly-housed PoWeR cohorts that underwent a 1-week introduction to an unweighted wheel, followed by eight weeks of weighted wheel running under the progression outlined in Fig. 1. Forelimb grip strength was assessed by a single NNMTi treatment-blinded investigator during week six and averaged across 2–4 trials/mouse. At the end of week 8, the strength of the right limb plantarflexor muscle complex was measured using an in vivo isometric peak tetanic torque technique. Animals were anesthetized using ~ 2.5% isoflurane, and the tibial nerve was stimulated. During this procedure, electrode placement was optimized to minimize antagonistic dorsiflexion. An ideal amperage was defined and maintained during a force-frequency experiment to establish peak tetanic torque. Immediately after, each mouse underwent a fatigue test of 54 submaximal contractions at 60 Hz. The peak torque of each contraction peak torque and the maximum for all contractions were computed. The number of contractions with peak torque values exceeding 50% and 70% of that maximum were defined for each mouse. Cumulative work was calculated by summing the integral of each contraction after correction for baseline force. Data for fatigue and cumulative work calculations were not recorded for three Sed cohort mice; additionally, two Sed cohort mice, one NNMTi-treated Sed mouse, and one NNMTi-treated PoWeR mouse exhibited cumulative workloads that suggested an inability to perform the task.

After the fatigue test, mice were euthanized, and tissues were weighed and collected. Of note, hind limb muscles from the right limb (the limb that underwent in vivo isometric peak tetanic torque and fatigue testing) were processed for immunohistochemistry to avoid acute effects of muscle functional testing on the proteome and metabolome. Hind limb muscles from the left limb that did not undergo torque and fatigue testing were flash-frozen for proteome and metabolome analyses. Given the terminal BW differences between the Sed and PoWeR groups and the potential for differences in the sarcopenic state of the animals, BWN values are emphasized. However, raw muscle masses are reported in Supplemental Table 1 for completeness. The solei of one Sed mouse and one NNMTi-treated PoWeR mouse were not collected.

Further details on housing conditions and protocols for in vivo and euthanasia methods are in Supplemental File 1.

### Immunohistochemistry

Flash-frozen right-limb plantarflexor muscles were cut on a cryostat, air-dried, and assessed for muscle fiber type distribution and type-specific CSA using Myovision (https://www.uky.edu/chs/center-for-muscle-biology/myovision#Download) and the reagents and equipment outlined in Supplemental Table 4 and methods of Supplemental File 1. Unstained myofibers were counted as Type IIx fibers. The percentage of each fiber type was missing for one PoWeR cohort mouse’s gastrocnemius; the plantaris fiber type and CSA data were missing from one PoWeR cohort mouse. The solei of one Sed mouse and one NNMTi-treated PoWeR mouse had missing immunohistochemical data in addition to missing masses.

Gastrocnemius IMCL content was assessed on fresh-cut (not air-dried), post-fixed samples, similar to our prior protocols^77^. Reagents and equipment are outlined in Supplemental Table 4, and additional protocol details are in Supplemental File 1. IMCL content was quantified with Zeiss Zen software (v3.1, https://www.zeiss.com/microscopy/en/products/software/zeiss-zen.html). Individual muscle fibers were manually delineated as regions of interest, and IMCL content was calculated as the average integrated density of fifty fibers of each type (smaller [oxidative] and larger [glycolytic]) per sample.

### -Omics sample preparation

To avoid altering molecular signaling in the gastrocnemius muscle right before collection, the contralateral limb (i.e., the limb not used for terminal torque and fatigue measurements) was immediately flash-frozen when the animals were euthanized, and it was this flash-frozen unstressed muscle that was used for -omics sample preparation. The gastrocnemius samples used for -omic analyses were processed in two batches, one with the Sed group samples (n = 8 Sed; n = 8 NNMTi-treated Sed) and the other with the PoWeR group samples (n = 7 PoWeR; n = 7 NNMTi-treated PoWeR). Reagents and equipment used for sample processing are outlined in Supplemental Table 4, with protocols outlined in Supplemental File 1. All samples were diluted to 2 mg/mL.

### Proteomics

Supplemental File 1 contains additional proteomics workflow and analysis details; reagents and equipment are in Supplemental Table 4; scripts are in Supplemental File 2. Samples were processed in two batches (Sed cohorts, then PoWeR cohorts). Notably, two samples (one Sed sample and one NNMTi-treated Sed sample) were analyzed in both batches to aid cross-batch comparisons. In brief, samples were TMTpro reagent-labeled, pooled, and separated into eight fractions before being analyzed in a mass spectrometer coupled to a liquid chromatography system using an MS3 method and positive ion mode. Raw data files were analyzed in Proteome Discoverer (v2.4, https://www.thermofisher.com/us/en/home/industrial/mass-spectrometry/liquid-chromatography-mass-spectrometry-lc-ms/lc-ms-software/multi-omics-data-analysis/proteome-discoverer-software.html; Thermo Fisher Scientific), with peptide identification performed using Sequest HT searching against the UniProt mouse protein database^78^. Reporter intensity was used for downstream analyses. Analysis of raw reporter intensities of the pre- and post-digestion controls supported reproducibility of the trypsin digestion process and technical reproducibility within and across batches with coefficients of variation (CVs) of 40% and 35% across all unique samples, respectively.

Raw MS3 data files were aggregated across batches; proteins with a zero value in a batch standard or in > 50% of a particular cohort’s samples were removed. The remaining missing values were imputed through a mixed imputation within each cohort. All samples were normalized by sample loading, trimmed mean of M values, and then internal reference scaling^79^ to the average of the two samples repeated across batches. Once normalized, differential expression was determined with EdgeR^80^. To increase the number of proteins available for cellular pathway and process analyses, DEPs discussed herein did not undergo correction for multiple comparisons. FDR-corrected *P* values are reported in Supplemental Table 2, where a Benjamini–Hochberg correction for multiple comparisons was used to acquire these values.

### Metabolomics

Supplemental File 1 includes additional metabolomic workflow and analysis details; reagents and equipment are in Supplemental Table 4. In brief, samples from all groups were simultaneously analyzed for metabolites and heavy isotope standards within a single set of mass spectroscopy experiments; mass spectrometrists were blinded to the NNMTi treatment cohort. One PoWeR control sample was excluded for issues during sample preparation of its aliquot. Experiments used the ESI ion spray source in positive and negative ion modes and hydrophilic interaction liquid chromatography. Data were processed by SCIEX MultiQuant (v3.0.3, https://sciex.com/products/software/multiquant-software) software with relative quantification based on each metabolite’s peak area. As noted in Supplemental Table 3, the CV was < 25% between quality control samples run at the beginning and end of the positive and negative ion mode mass spectrometric runs, suggesting the system was working correctly. Additionally, the CVs for the heavy isotope standards remained under 15% for both runs, reinforcing that there were no issues throughout the ionization process.

The approach to determining DEMs was similar to that for DEPs. DEMs discussed herein were significant before correction for multiple comparisons; FDR-corrected *P* values are reported in Supplemental Table 3. Quantifications from the negative and positive polarity column analyses are reported for metabolites; when relevant (i.e., glutathione, S-adenosyl-L-homocysteine), both results are reported since these were processed separately.

### Statistics and software

Non-omics statistics were run in GraphPad Prism (v9, www.graphpad.com) and explored main effects of exercise, NNMTi treatment, and an interaction; additional statistical methods details are in Supplemental File 1. In brief, physiological data that did not include repeated measures were tested for normality and heteroscedasticity and processed accordingly with the appropriate parametric or non-parametric test after transformation. When possible, only Sed NNMTi vs Sed, PoWeR NNMTi vs PoWeR, and Sed vs PoWeR comparisons were run and only these are reported in the figures. For fiber-type analyses, when applicable, zeroes were included in the statistics for percentages. If the fiber type was absent, the data for CSA calculations were considered missing (not zero).

A Geisser-Greenhouse correction was applied to repeated measures data since sphericity was never assumed; a restricted maximum likelihood mixed effects model was used if data points were missing. Molecular functions were mapped using Gorilla^81^, with the DEPs input as UniProt IDs and the background list as all 2512 proteins analyzed (last ran October 7, 2022); protein class analysis was performed UniProt IDs in PANTHER (v17.0, https://pantherdb.org/)^82,83^, and data are reported in Supplemental Fig. 3; overlaying Venn diagrams were generated with BioVenn^84^; the chord plot was generated with Goplot^85^.

### Supplementary Information

Supplementary Figures.

Supplementary Tables.

Supplementary Information 1.

Supplementary Information 2.

## Data Availability

Datasets generated and analyzed through this work are available in supplemental files and raw proteomics files are available at MassIVE under dataset identifier MSV000094700, while raw metabolomics files are available at MetaboLights under study identifier MTBLS10116.
