# Supplementary material for: Nicotinamide N-methyltransferase inhibition mimics and boosts exercise-mediated improvements in muscle function in aged mice
Source: Sci Rep. 2024 Jul 5;14:15554. doi: 10.1038/s41598-024-66034-9 (PMC11226645; doi:10.1038/s41598-024-66034-9)
Supplement: Supplementary file 1 — Supplementary Figures. [file 41598_2024_66034_MOESM1_ESM.pptx]

## Slide 1
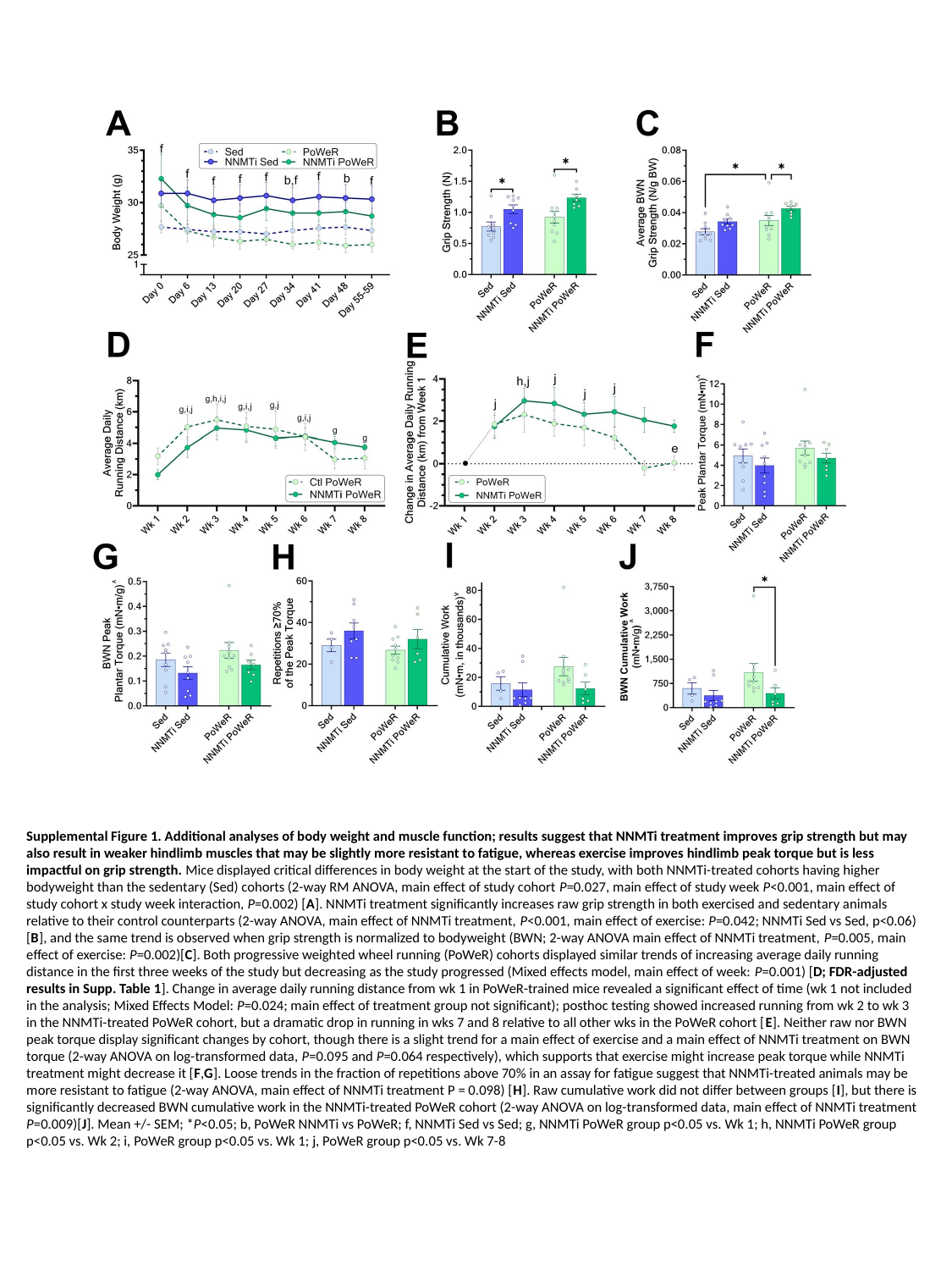

Supplemental Figure 1. Additional analyses of body weight and muscle function; results suggest that NNMTi treatment improves grip strength but may also result in weaker hindlimb muscles that may be slightly more resistant to fatigue, whereas exercise improves hindlimb peak torque but is less impactful on grip strength. Mice displayed critical differences in body weight at the start of the study, with both NNMTi-treated cohorts having higher bodyweight than the sedentary (Sed) cohorts (2-way RM ANOVA, main effect of study cohort P=0.027, main effect of study week P<0.001, main effect of study cohort x study week interaction, P=0.002) [A]. NNMTi treatment significantly increases raw grip strength in both exercised and sedentary animals relative to their control counterparts (2-way ANOVA, main effect of NNMTi treatment, P<0.001, main effect of exercise: P=0.042; NNMTi Sed vs Sed, p<0.06) [B], and the same trend is observed when grip strength is normalized to bodyweight (BWN; 2-way ANOVA main effect of NNMTi treatment, P=0.005, main effect of exercise: P=0.002)[C]. Both progressive weighted wheel running (PoWeR) cohorts displayed similar trends of increasing average daily running distance in the first three weeks of the study but decreasing as the study progressed (Mixed effects model, main effect of week: P=0.001) [D; FDR-adjusted results in Supp. Table 1]. Change in average daily running distance from wk 1 in PoWeR-trained mice revealed a significant effect of time (wk 1 not included in the analysis; Mixed Effects Model: P=0.024; main effect of treatment group not significant); posthoc testing showed increased running from wk 2 to wk 3 in the NNMTi-treated PoWeR cohort, but a dramatic drop in running in wks 7 and 8 relative to all other wks in the PoWeR cohort [E]. Neither raw nor BWN peak torque display significant changes by cohort, though there is a slight trend for a main effect of exercise and a main effect of NNMTi treatment on BWN torque (2-way ANOVA on log-transformed data, P=0.095 and P=0.064 respectively), which supports that exercise might increase peak torque while NNMTi treatment might decrease it [F,G]. Loose trends in the fraction of repetitions above 70% in an assay for fatigue suggest that NNMTi-treated animals may be more resistant to fatigue (2-way ANOVA, main effect of NNMTi treatment P = 0.098) [H]. Raw cumulative work did not differ between groups [I], but there is significantly decreased BWN cumulative work in the NNMTi-treated PoWeR cohort (2-way ANOVA on log-transformed data, main effect of NNMTi treatment P=0.009)[J]. Mean +/- SEM; *P<0.05; b, PoWeR NNMTi vs PoWeR; f, NNMTi Sed vs Sed; g, NNMTi PoWeR group p<0.05 vs. Wk 1; h, NNMTi PoWeR group p<0.05 vs. Wk 2; i, PoWeR group p<0.05 vs. Wk 1; j, PoWeR group p<0.05 vs. Wk 7-8

## Slide 2
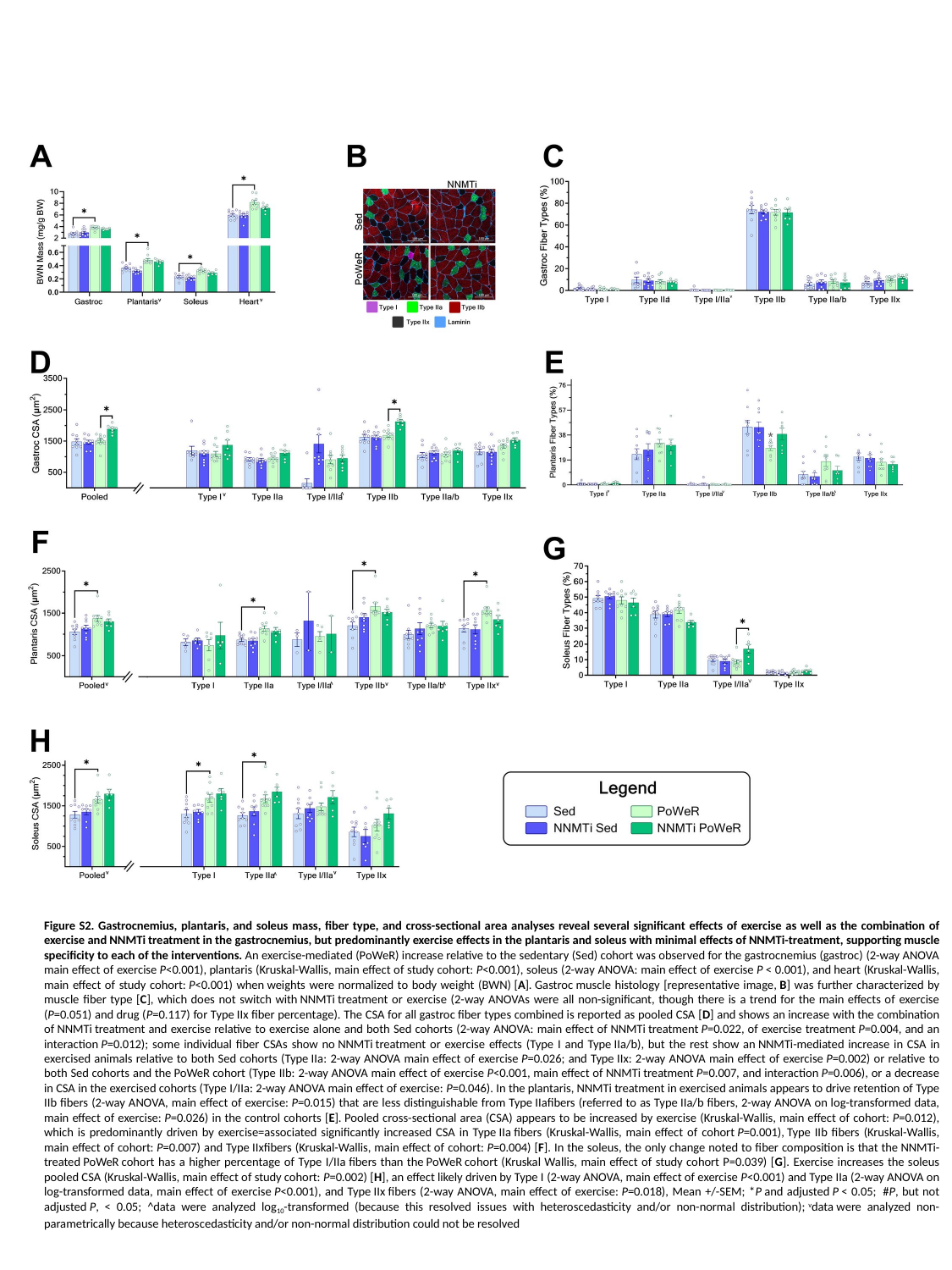

Figure S2. Gastrocnemius, plantaris, and soleus mass, fiber type, and cross-sectional area analyses reveal several significant effects of exercise as well as the combination of exercise and NNMTi treatment in the gastrocnemius, but predominantly exercise effects in the plantaris and soleus with minimal effects of NNMTi-treatment, supporting muscle specificity to each of the interventions. An exercise-mediated (PoWeR) increase relative to the sedentary (Sed) cohort was observed for the gastrocnemius (gastroc) (2-way ANOVA main effect of exercise P<0.001), plantaris (Kruskal-Wallis, main effect of study cohort: P<0.001), soleus (2-way ANOVA: main effect of exercise P < 0.001), and heart (Kruskal-Wallis, main effect of study cohort: P<0.001) when weights were normalized to body weight (BWN) [A]. Gastroc muscle histology [representative image, B] was further characterized by muscle fiber type [C], which does not switch with NNMTi treatment or exercise (2-way ANOVAs were all non-significant, though there is a trend for the main effects of exercise (P=0.051) and drug (P=0.117) for Type IIx fiber percentage). The CSA for all gastroc fiber types combined is reported as pooled CSA [D] and shows an increase with the combination of NNMTi treatment and exercise relative to exercise alone and both Sed cohorts (2-way ANOVA: main effect of NNMTi treatment P=0.022, of exercise treatment P=0.004, and an interaction P=0.012); some individual fiber CSAs show no NNMTi treatment or exercise effects (Type I and Type IIa/b), but the rest show an NNMTi-mediated increase in CSA in exercised animals relative to both Sed cohorts (Type IIa: 2-way ANOVA main effect of exercise P=0.026; and Type IIx: 2-way ANOVA main effect of exercise P=0.002) or relative to both Sed cohorts and the PoWeR cohort (Type IIb: 2-way ANOVA main effect of exercise P<0.001, main effect of NNMTi treatment P=0.007, and interaction P=0.006), or a decrease in CSA in the exercised cohorts (Type I/IIa: 2-way ANOVA main effect of exercise: P=0.046). In the plantaris, NNMTi treatment in exercised animals appears to drive retention of Type IIb fibers (2-way ANOVA, main effect of exercise: P=0.015) that are less distinguishable from Type IIafibers (referred to as Type IIa/b fibers, 2-way ANOVA on log-transformed data, main effect of exercise: P=0.026) in the control cohorts [E]. Pooled cross-sectional area (CSA) appears to be increased by exercise (Kruskal-Wallis, main effect of cohort: P=0.012), which is predominantly driven by exercise=associated significantly increased CSA in Type IIa fibers (Kruskal-Wallis, main effect of cohort P=0.001), Type IIb fibers (Kruskal-Wallis, main effect of cohort: P=0.007) and Type IIxfibers (Kruskal-Wallis, main effect of cohort: P=0.004) [F]. In the soleus, the only change noted to fiber composition is that the NNMTi-treated PoWeR cohort has a higher percentage of Type I/IIa fibers than the PoWeR cohort (Kruskal Wallis, main effect of study cohort P=0.039) [G]. Exercise increases the soleus pooled CSA (Kruskal-Wallis, main effect of study cohort: P=0.002) [H], an effect likely driven by Type I (2-way ANOVA, main effect of exercise P<0.001) and Type IIa (2-way ANOVA on log-transformed data, main effect of exercise P<0.001), and Type IIx fibers (2-way ANOVA, main effect of exercise: P=0.018), Mean +/-SEM; *P and adjusted P < 0.05;  #P, but not adjusted P, < 0.05; ^data were analyzed log10-transformed (because this resolved issues with heteroscedasticity and/or non-normal distribution); vdata were analyzed non-parametrically because heteroscedasticity and/or non-normal distribution could not be resolved

## Slide 3
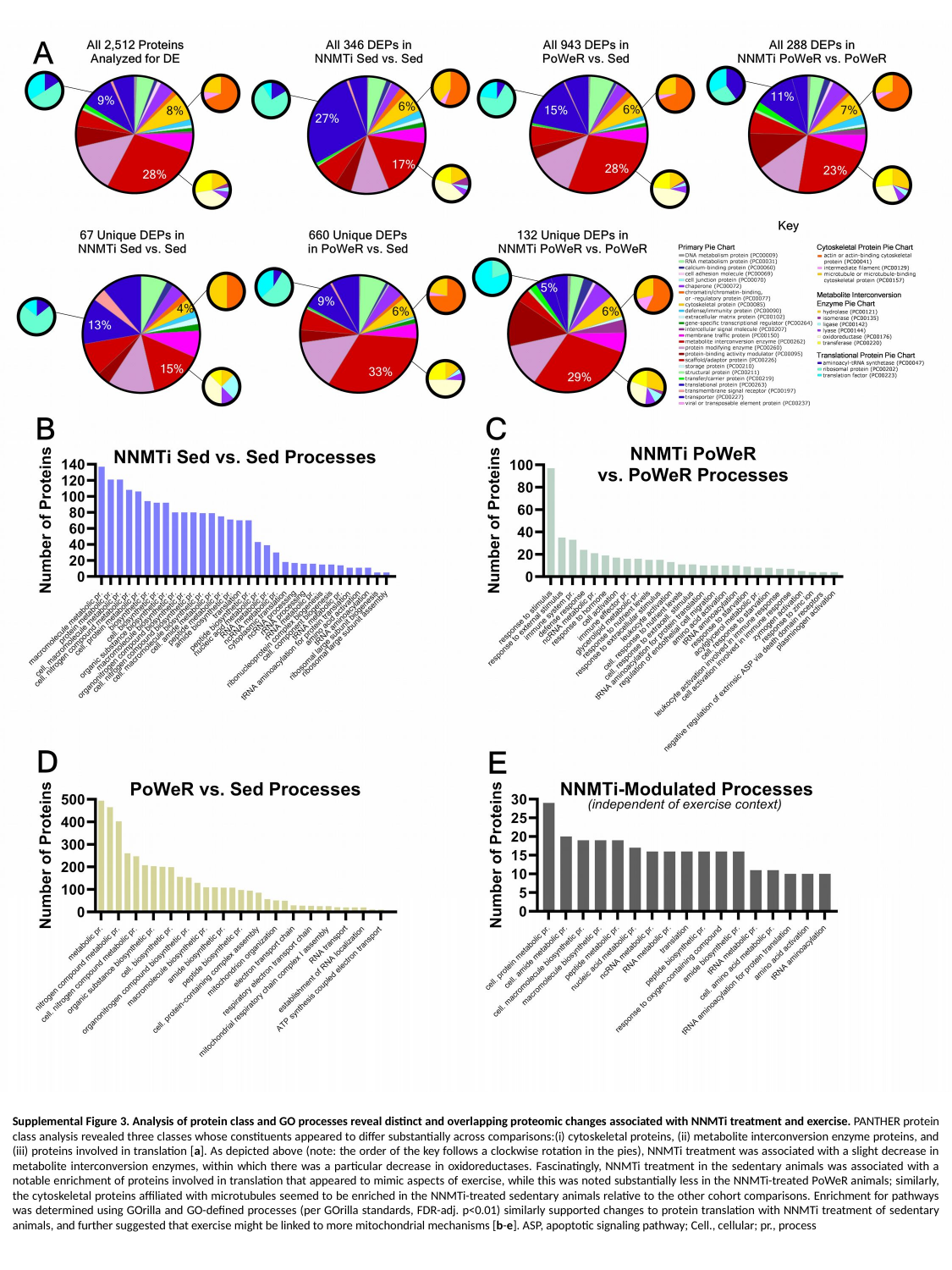

Supplemental Figure 3. Analysis of protein class and GO processes reveal distinct and overlapping proteomic changes associated with NNMTi treatment and exercise. PANTHER protein class analysis revealed three classes whose constituents appeared to differ substantially across comparisons:(i) cytoskeletal proteins, (ii) metabolite interconversion enzyme proteins, and (iii) proteins involved in translation [a]. As depicted above (note: the order of the key follows a clockwise rotation in the pies), NNMTi treatment was associated with a slight decrease in metabolite interconversion enzymes, within which there was a particular decrease in oxidoreductases. Fascinatingly, NNMTi treatment in the sedentary animals was associated with a notable enrichment of proteins involved in translation that appeared to mimic aspects of exercise, while this was noted substantially less in the NNMTi-treated PoWeR animals; similarly, the cytoskeletal proteins affiliated with microtubules seemed to be enriched in the NNMTi-treated sedentary animals relative to the other cohort comparisons. Enrichment for pathways was determined using GOrilla and GO-defined processes (per GOrilla standards, FDR-adj. p<0.01) similarly supported changes to protein translation with NNMTi treatment of sedentary animals, and further suggested that exercise might be linked to more mitochondrial mechanisms [b-e]. ASP, apoptotic signaling pathway; Cell., cellular; pr., process

## Slide 4
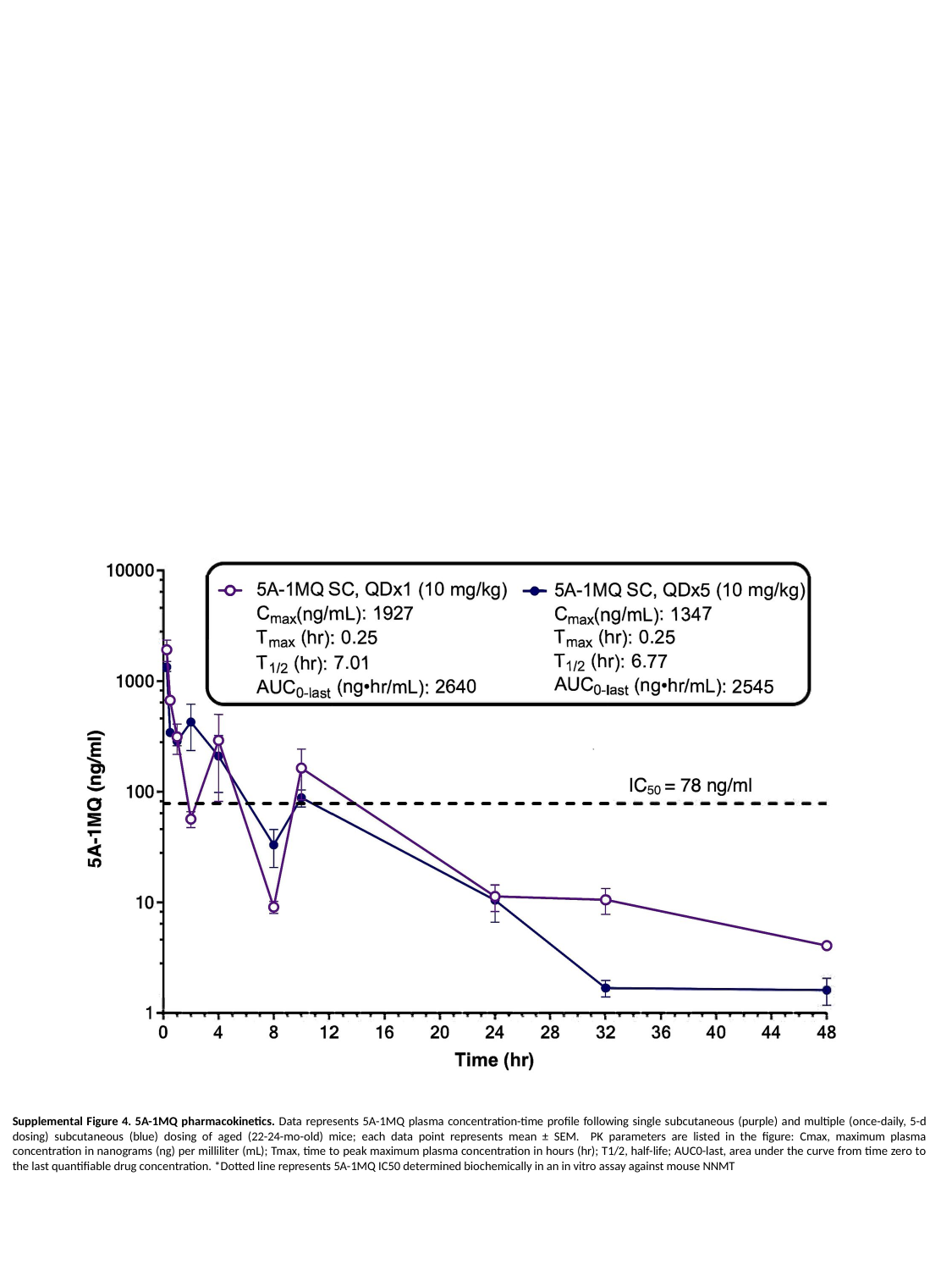

Supplemental Figure 4. 5A-1MQ pharmacokinetics. Data represents 5A-1MQ plasma concentration-time profile following single subcutaneous (purple) and multiple (once-daily, 5-d dosing) subcutaneous (blue) dosing of aged (22-24-mo-old) mice; each data point represents mean ± SEM. PK parameters are listed in the figure: Cmax, maximum plasma concentration in nanograms (ng) per milliliter (mL); Tmax, time to peak maximum plasma concentration in hours (hr); T1/2, half-life; AUC0-last, area under the curve from time zero to the last quantifiable drug concentration. *Dotted line represents 5A-1MQ IC50 determined biochemically in an in vitro assay against mouse NNMT
