# Supplementary material for: Nicotinamide N-methyltransferase inhibition mimics and boosts exercise-mediated improvements in muscle function in aged mice
Source: Sci Rep. 2024 Jul 5;14:15554. doi: 10.1038/s41598-024-66034-9 (PMC11226645; doi:10.1038/s41598-024-66034-9)
Supplement: Supplementary file 3 — Supplementary Information 1. [file 41598_2024_66034_MOESM3_ESM.pdf]

Methods

Independent Pharmacokinetic Study Methods

The pharmacokinetic study for RT001 in **Supplemental Figure 4** was performed at WuXi AppTec (Cranbury, NJ); animals were acclimated at least 3 days, group housed in a temperature- and humidity-controlled environment (20-26°C, 30-70% relative humidity), and maintained on a 12-hour light/dark cycle with food (Diet #5002, LabDiet) and water (reverse osmosis) available *ad libitum*. In a cohort of male 22-24-month-old C57BL/6 mice (Hilltop Lab Animals, Inc.), blood samples were serially collected after single subcutaneous dosing of RT001 at 10mg/kg dose, or 5 days of once-daily subcutaneous dosing of RT001 at 10mg/kg dose. Each mouse had five samples collected from it, with a minimum 45-minute gap between collections; blood was collected from the saphenous vein into pre-chilled K_2EDTA-coated tubes and stored on ice until centrifugation at 4°C, 3000g for 5 minutes within 30 minutes of collection. Plasma was stored at -70°C $\pm$ 10°C until analysis with liquid chromatography tandem mass spectrometry (LC-MS/MS). N=3 samples were collected at all time points (0.25, 0.5, 1, 2, 4, 8, 10, 24, 32, and 48 hours post-dose), except n=2 were collected for 0.5, 2, 8, 24, and 48 hours post-dose for singly-dosed animals; one sample was below the limit of detection at 48 hours post-dose in multiple-dosed animals and was therefore excluded from the plot and calculations.

Animals & Ethical Approval

Animal procedures were performed according to the appropriate guidelines detailed in the manuscript. The US NIH National Institute on Aging Aged Rodent Colony is maintained at Charles River Laboratories. Mice were housed in the same temperature and humidity-controlled room on a 12h:12h light-dark cycle with *ad libitum* access to food (Teklad Irradiated Global 18% Protein Rodent Diet, catalog #2918) and water; food consumption was not monitored throughout this study. Following arrival, mice were acclimated for at least seven days before experiments began.

Mice in the PoWeR group were singly housed in cages with running wheels to allow for monitoring of individual running volume using ClockLab software (Actimetrics v6.1.01, https://actimetrics.com/products/clocklab/). Mice in the Sed group were group housed in cages without running wheels. The PoWeR groups had an introductory week with an unweighted wheel; due to experimental constraints, the volume of running during this introductory week was not recorded. After the introductory week, mice had 8 weeks of voluntary wheel running with two, three, and four grams total weight added to the wheels at the start of weeks 1, 2, and 3, respectively. At the beginning of week 4, five grams of total weight were added to each wheel, and these remained in place through week 8. One g magnets were affixed to one side of the wheel to allow for the progressive increase in weight [1]. A limitation of the wheel running being voluntary is mouse-to-mouse variability in running distance, as was noted in this study, with most animals running less than 6 km/day on average across the 8 weeks but two animals running an average of >8 km/day. During week 6 of the study, grip strength was assessed by a single investigator holding each mouse at the base of the tail and placing the forelimb paws pronated on the horizontal bar of the grip strength apparatus. Mice were held horizontally, preventing hindlimbs from interfering, and gently pulled away from the apparatus until both

forelimb paws released their grip. The maximal force (Newtons) achieved for 2-4 trials was averaged.

Following 8 weeks of NNMTi or vehicle treatment, contractile function and fatiguability of the plantarflexors were measured on the right hindlimb. Then mice were humanely euthanized by cervical dislocation under deep anesthesia with inhaled isoflurane. Right hindlimb plantarflexor muscles (*i.e.*, soleus, plantaris, gastrocnemius) were rapidly collected, weighed, and processed for immunohistochemical analyses. Left hindlimb muscles were collected and flash-frozen immediately for proteomics and metabolomics.

Plantar Flexion Peak Torque and Fatigue

After 8 weeks of study, the strength of the plantarflexor muscle complex was measured in the right hindlimb of all animals using *in vivo* isometric peak tetanic torque. In an induction chamber, mice were anesthetized with 2.5% isoflurane vaporized in 1.5 L/min oxygen. Mice were then transferred to a secure nose cone with a continuous flow of isoflurane in oxygen. The right hindlimb was analyzed for all mice, with fur trimmed to ensure unobstructed electrode placement. Mice were placed in the supine position on a 37°C temperature-regulated platform, and the hindlimb secured using a clamp at the knee with the foot placed in a footplate on a dual-mode lever and motor. Surgical tape was wrapped around the foot to secure it to the footplate and prevent heel movement. The footplate and motor arm were adjusted to place the tibia parallel to the platform with a 90-degree angle at the ankle. Needle electrodes were positioned percutaneously slightly lateral to the knee to maximally stimulate the tibial nerve using an electrical stimulator. Using repeated twitches with the Instant Stimulation function with Live View in Dynamic Muscle Control LabBook (DMC v6.000, https://aurorascientific.com/products/muscle-physiology/muscle-daq-software/605a-dynamic-muscle-system/), the placement of needle electrodes was adjusted to optimize location to generate maximum isometric torque and eliminate antagonistic dorsiflexion. The optimal amperage to produce maximal torque was determined by a progressive series of twitch experiments (0.05s stimulus duration) beginning with 10 milliAmp and increasing in small increments until the maximal torque stimulated by the minimum amperage was recorded; this amperage was then maintained throughout the force-frequency experiment (10, 40, 80, 120, 150, 180, and 200Hz; 0.25s stimulus duration with a 2min rest period between each stimulus) from which isometric peak tetanic torque was recorded. Data were collected using DMC v6.000 and analyzed with Dynamic Muscle Analysis software (DMA v5.501, https://aurorascientific.com/products/muscle-physiology/muscle-daq-software/605a-dynamic-muscle-system/; software and hardware are integrated using the 3-in-1 Whole Animal System, https://aurorascientific.com/products/muscle-physiology/systems/1300a-whole-animal-system-mice/).

Upon completing the force-frequency curve, animals underwent another twitch test then each mouse underwent a fatigue test of 54 submaximal contractions at 60Hz. Each contraction was stored as a text file containing 7,500 values (750ms total sampling time, 0.1ms sampling rate). Each animal's text files were queried in Matlab (R2022a, script in **File S2**) to determine the peak torque for each contraction; the 54 values for each animal were stored together in a vector.

The maximum value within the vector was multiple by 0.5 or 0.7, representing 50% and 70% of the peak torque, respectively. The number of repetitions equal to or exceeding the respective 50% and 70% values were determined. To determine cumulative work, the integral of each text file was calculated, with a correction for baseline force measured by the transducer, and the sum of these 54 integrals computed for each animal. Finally, as an exploratory measure, animals underwent one more plantarflexor torque and twitch test and then were euthanized.

Immunohistochemistry

Dissected muscles from mice were mounted in Tissue Tek O.C.T. Compound at resting length, frozen in liquid nitrogen-cooled 2-methylbutane, and stored at −80°C until analysis.

For immunofluorescent measurement of muscle fiber type distribution and fiber type-specific CSA, 7µm-thick sections were cut with a cryostat and allowed to air dry for 1 h before storage at −20°C. Sections were then blocked for 1h at room temperature in a mouse-on-mouse IgG blocking solution, followed by a PBS wash. Slides were incubated overnight at 4°C in primary antibodies against laminin (rabbit polyclonal, 1:200) and myosin heavy chain (MHC) Type I (mouse IgG2b, 1:100), MHC Type IIa (mouse IgG1) and MHC type IIb (mouse IgM) supernatants. The next day, slides were washed in PBS and incubated for 90 min at room temperature in the following secondary antibodies in PBS: goat anti-rabbit IgG AF350 (1:250), goat anti-mouse IgG2b AF647 (1:250), goat anti-mouse IgG1 AF488 (1:500), and goat anti-mouse IgM AF555 (1:500). Slides were washed in PBS, followed by post-fixation for 3 min in methanol and another wash in PBS. Slides were mounted using Vectashield fluorescence mounting media. Images were captured at 100x magnification with a Zeiss upright microscope.

For the determination of intramuscular lipid (IMCL) content, fresh-cut (without air drying) slides from each study animal were fixed in 4% PFA for 7 min at room temperature, similar to our prior protocols [2]. Sections were then permeabilizated in 0.5% Triton-X100 in PBS for 10min. Slides were stained with BODIPY (4,4-Difluoro-1,3,5,7,8-Pentamethyl-4-Bora-3a,4a-Diaza-s-Indacenep) and Wheat Germ Agglutinin AF594 conjugate before being mounted with fluorescent mounting media. Slides were kept in a dark container and protected from light exposure until imaging, and the time between mounting and imaging was kept consistent between samples. Whole cross-sectional images of the gastrocnemius muscle were captured at 100X total magnification using the tiles and stitching functions on the Zeiss microscope. Individual muscle fibers were manually delineated as individual regions of interest, and an intensity threshold was uniformly selected and applied to represent a positive signal for IMCL droplets.

-Omics Tissue Sample Processing

As mentioned in the manuscript, the samples used for -omic analyses were processed in two separate batches. In both instances, samples of flash-frozen gastrocnemius were diced and placed in RIPA buffer with protease inhibitors and a 7mm metal bead in each tube. Samples were homogenized using a Qiagen TissueLyser for 10min at 30Hz, then chilled for 20min at 4°C. Samples were pelleted through centrifugation at 4°C for 10min, supernatants were collected,

and their protein concentrations were determined using a bicinchoninic acid assay. Supernatants were diluted to 2 mg/mL with RIPA buffer (containing protease inhibitors at the same concentrations as before). Samples were then split into aliquots for subsequent proteomic and metabolomic analyses, and 150ng of recombinant hepatitis B surface antigen adw protein was added to each 50µL aliquot as a pre-digestion control.

Proteomics

As mentioned in the manuscript, the proteins of the homogenized samples were further processed and analyzed in two separate batches: one with samples from the Sed and NNMTi-treated Sed cohorts, and another with samples from the PoWeR and NNMTi-treated PoWeR cohorts. After the initial processing, SDS was added to a final concentration of 5%, with 50mM triethylammonium bicarbonate (TEAB) used as a diluent. DTT was added to a final concentration of 10mM, then samples were incubated at 56°C for 30min, cooled, and iodoacetamide was added to a final concentration of 20mM. Samples were incubated for 30min at room temperature in the dark, centrifuged for 2min at 13.2krpm, and supernatants collected. 50µL of each supernatant were digested overnight with trypsin at 37°C using an S-Trap. After digestion, the peptide eluate was dried and reconstituted in 100 mM triethylammonium bicarbonate buffer. Equal amounts of peptide (20-30µg) were taken from each sample, and 500fmol of beta-galactosidase was added as a post-digestion control and an internal standard to monitor liquid chromatography with tandem mass spectrometry (LC-MS/MS) reproducibility. Each sample in a respective batch was labeled with TMTpro reagent, giving it a unique identifier, then quenched with 5% hydroxylamine; after this, the samples for a respective batch were pooled. The pooled sample was fractionated into eight fractions using a reversed-phase fractionation spin column according to the manufacturer's instructions. The fractions were dried in a SpeedVac and reconstituted in a 2% acetonitrile, 0.1% TFA buffer.

Fractions were injected into an Orbitrap Fusion Lumos mass spectrometer coupled to an Ultimate 3000 RSLC-Nano liquid chromatography system. Samples were injected onto a 75 um i.d., 75-cm long EasySpray column and eluted with a gradient from 0-28% buffer B over 180min. Buffer A contained 2% (v/v) ACN and 0.1% formic acid in water, and buffer B had 80% (v/v) ACN, 10% (v/v) trifluoroethanol, and 0.1% formic acid in water. Samples were analyzed with an MS3 method, and the mass spectrometer operated in positive ion mode with a source voltage of 1.8kV and an ion transfer tube temperature of 275°C. MS scans were acquired at 120,000 resolution in the Orbitrap, and top speed mode was used for SPS-MS3 analysis with a cycle time of 3s. The top 10 fragments were selected for MS3 fragmentation using higher-energy C-trap dissociation, with a collision energy of 55%. Dynamic exclusion was set for 25s after an ion was selected for fragmentation.

As mentioned elsewhere, raw MS data files were analyzed using Proteome Discoverer (v2.4 https://www.thermofisher.com/us/en/home/industrial/mass-spectrometry/liquid-chromatography-mass-spectrometry-lc-ms/lc-ms-software/multi-omics-data-analysis/proteome-discoverer-software.html ; Thermo Fisher Scientific), with peptide identification performed using Sequest HT searching against the mouse protein database from UniProt. Fragment and precursor tolerances of 10ppm and 0.6Da were specified, and three

missed cleavages were allowed. Carbamidomethylation of Cys and TMTpro labeling of N-termini and Lys sidechains were set as fixed modifications, with oxidation of Met set as a variable modification. The false-discovery rate (FDR) cutoff was 1% for all peptides.

Pre- and post-digestion controls were analyzed separately and removed before downstream processing since they had been spiked in. The MS3 raw data files were processed as follows: First, each MS3 file was aggregated across batches to include only proteins identified in both batches, and the two samples repeated across the batches (referred to hereafter as 'batch standards') were averaged for each batch and added to the dataset; for the two Sed animal samples that were repeated with the PoWeR samples in batch 2, the average was retained as the batch standard, but the individual values were dropped from the dataset. At this point, less than 3% of the data were missing. Seven proteins with a zero value in one of the batch standards were removed from the data to prevent floor effects from being applied to the normalization process and avoid reliance on averaging to just one batch standard. Additionally, two protein IDs (A0A140T8M5 and O35490) for which >50% of the samples in a particular cohort had a value of zero were removed since the reduced number of measurements might contribute to a reduced variance for that specific cohort and could, in turn, increase the probability of a false positive. After this initial data cleaning, there were no instances where >2 samples were equal to zero in one cohort and only five instances where two samples had zero values across multiple cohorts. The protein ID lists were run through UniProt's Retrieve/ID mapping tool [3] to identify obsolete entries; none were identified. Within each cohort, we ran a mixed imputation that utilized the k-nearest neighbor method for data missing at random and the zero method for data missing not at random (script provided in **File S2**). Because our script derived from the DEP R package (v 1.22.0, https://www.bioconductor.org/packages/release/bioc/html/DEP.html) [4] and consequently values were log scaled during imputation, we reversed the log scaling and compiled all study cohort data together.

After imputation, samples were normalized by sample loading (importantly, the batch standards were not included in the computation of the global scaling value), followed by trimmed mean of M values (the reference column for which was not allowed to be one of the batch standards), and finally, internal reference scaling [5] to the average of the two samples repeated across batches (script provided **File S2**). This series of steps allowed for cross-comparison across batches. Once normalized, the data were compared using EdgeR (the data were fit to a quasi-likelihood negative binomial generalized linear model, and empirical Bayes quasi-likelihood F-tests were used to determine differential expression [6]; EdgeR v3.43.7, https://bioconductor.org/packages/release/bioc/html/edgeR.html). As noted elsewhere, a Benjamini-Hochberg correction for multiple comparisons was used to acquire FDR-adjusted p values, but data reported as significant in the manuscript text was significant before correction and may not meet statistical significance post-correction. This study focused on three comparisons of interest: NNMTi-treated Sed vs. Sed, NNMTi-treated PoWeR vs. PoWeR, and PoWeR vs. Sed.

Metabolomics

Metabolomic analysis used the aliquot set aside during the initial processing of the gastrocnemius muscle (discussed above; one PoWeR control sample was excluded for issues during sample preparation of that aliquot). Though the PoWeR and Sed cohort samples had been separately prepped, for the metabolome all groups were simultaneously analyzed for metabolites within a respective mass spectroscopy experiment, thus no monitoring for batch variation was needed. Samples were diluted 5-fold in chilled 100% high-performance liquid chromatography [HPLC]-grade methanol (final concentration of ~30mg of tissue per mL), vortexed 1 min at 4°C, incubated for 2 hours at -80°C, and centrifuged at 14,000xg for 15min at 4°C. Sample supernatants were transferred to a new tube ("supernatant #1"). The remaining pellet was resuspended in 400µl of 80% (v/v) methanol (cooled to −80°C), vortexed for 1min at 4°C, incubated for 30min at −80°C, and centrifuged at 14,000xg for 15min at 4°C; supernatant from this procedure was added to supernatant #1 and this pooled sample stored at -80°C. The pooled supernatants were then thawed, filtered through a 0.22µM polyvinylidene fluoride (PVDF) filter, and 100µL of the filtered supernatant speed vacuumed to a pellet at room temperature (without heat) for 50min. The pellets were re-suspended in a 100µL of 80/20 (v/v) methanol/water (liquid chromatography-mass spectrometry-grade) and 0.2µM heavy isotope (^{13}C) standards added with vortexing. The samples were placed randomly into the LC/MS-MS autosampler for analysis

Mass spectrometric analyses were performed on a SCIEX "QTRAP 6500+" mass spectrometer, which detected the parent and daughter ions. The mass spectrometer was equipped with an electrospray ionization (ESI) ion spray source coupled to a Shimadzu HPLC (Nexera X2 LC-30AD); Analyst (v1.7.1, https://sciex.com/products/software/analyst-software; SCIEX) software controlled the system. The ESI source was used in both positive and negative ion modes. The ion spray needle voltages used for multiple reaction monitoring (MRM) positive and negative polarity modes were set at 4800V and −4000V, respectively.

Chromatography was performed under hydrophilic interaction liquid chromatography (HILIC) conditions using a SeQuant® ZIC®-pHILIC 5µm polymeric 150×2.1mm polyetheretherketone (PEEK)-coated HPLC column. The column temperature, sample injection volume, the flow rate was set to 45°C, 5µL, and 0.15 mL/min, respectively. The HPLC conditions used 20 mM ammonium carbonate, including 0.1% ammonium hydroxide, as solvent A and acetonitrile as solvent B, with a gradient of 80% solvent B at 0min, 20% solvent B at 20 min, 80% solvent B at 20.5min, and 80% solvent B at 34min.

The approach used to determine DEMs was similar to that used for proteomic analysis, except that batch standards and internal reference scaling were not required. Metabolites identified with the positive polarity column and those identified with the negative polarity column were processed separately since these are separate runs with different methodologies. Still, given that the same samples were run, they would not be considered batches. Imputation was not needed for any metabolites analyzed with the negative polarity column but was necessary for a single metabolite in the NNMTi-treated Sed cohort. As with the proteomics dataset, this value was imputed similarly, then the log scaling was reversed, and the value replaced the original zero in the dataset. The metabolomics datasets (positive and negative polarity column datasets,

respectively) were adjusted using sample loading normalization and trimmed mean of M values. Differential expression was determined using EdgeR with the Benjamini-Hochberg correction for multiple comparisons focused on the same three comparisons of interest.

Statistics and Software

Normally distributed and homoscedastic data were processed using traditional parametric tests; normally distributed and heteroscedastic data were processed using the appropriate parametric test accounting for the heteroscedasticity. A non-parametric test was used if a $\log_{10}$ transformation (or $\log_{10}$ transformation following a +1 transform in the instance any values were < 1) could not render the data normal and homoscedastic. The repeated measures data were not assessed for normality and heteroscedasticity since there is no conventional nonparametric equivalent to the two-way repeated measures analysis of variance (ANOVA). Results were adjusted for multiple comparisons using the two-stage linear step-up procedure of Benjamini, Krieger and Yekutieli.

References

1 Dungan, C. M. *et al.* Elevated myonuclear density during skeletal muscle hypertrophy in response to training is reversed during detraining. *Am J Physiol Cell Physiol* **316**, C649-C654, doi:10.1152/ajpcell.00050.2019 (2019).

2 Moro, T., Brightwell, C. R., Volpi, E., Rasmussen, B. B. & Fry, C. S. Resistance exercise training promotes fiber type-specific myonuclear adaptations in older adults. *J Appl Physiol (1985)* **128**, 795-804, doi:10.1152/japplphysiol.00723.2019 (2020).

3 Consortium, U. UniProt: the universal protein knowledgebase in 2021. *Nucleic Acids Res* **49**, D480-D489, doi:10.1093/nar/gkaa1100 (2021).

4 Zhang, X. *et al.* Proteome-wide identification of ubiquitin interactions using UbIA-MS. *Nat Protoc* **13**, 530-550, doi:10.1038/nprot.2017.147 (2018).

5 Plubell, D. L. *et al.* Extended Multiplexing of Tandem Mass Tags (TMT) Labeling Reveals Age and High Fat Diet Specific Proteome Changes in Mouse Epididymal Adipose Tissue. *Molecular & cellular proteomics : MCP* **16**, 873-890, doi:10.1074/mcp.M116.065524 (2017).

6 Robinson, M. D., McCarthy, D. J. & Smyth, G. K. edgeR: a Bioconductor package for differential expression analysis of digital gene expression data. *Bioinformatics* **26**, 139-140, doi:10.1093/bioinformatics/btp616 (2010).
