# Supplementary material for: Nicotinamide N-methyltransferase inhibition mimics and boosts exercise-mediated improvements in muscle function in aged mice
Source: Sci Rep. 2024 Jul 5;14:15554. doi: 10.1038/s41598-024-66034-9 (PMC11226645; doi:10.1038/s41598-024-66034-9)

Supplemental File 2

R script to perform Metabolomics_Step1_ImputationForSingleMissingValue

```
#Install packages as needed
install.packages("reader")
install.packages("DEP")
install.packages("dplyr")
install.packages("tidyr")
install.packages("purrr")
install.packages("ggplot2")
install.packages("SummarizedExperiment")

#Load the packages into R
library("readr")
library("DEP")
library("tidyr")
library("purrr")
library("ggplot2")
library("SummarizedExperiment")
library("dplyr")

#Import the dataset
metabolomicsdata <- read_tsv("[path to file]")

#Get the columns that have the samples' data (this is set up assuming you have lots of
columns and only some of them are samples)
Sample_columns <- grep("Sample", colnames(metabolomicsdata))

#Layout how the data is formatted; e.g., which samples below to which condition (in this
case all are NNMTi_Sed), how many replicates)
#Note: This replicate number will be used later to generate a new name for the samples,
so it's best to keep the samples in an organized order (such as chronological) so that it's
clear which sample becomes 1, 2, 3, etc)
experimental_design <- data_frame(
  label = colnames(metabolomicsdata)[!colnames(metabolomicsdata) %in% c("name",
"ID")],
  condition = c(
    rep("NNMTi_Sed", 8)),

  replicate = rep(1:8))

#Make SummarizedExperiment object
data_se <- make_se(metabolomicsdata, Sample_columns, experimental_design)

#Grab the samples' data for the treatment group of interest
NNMTi_Sed <- grep("NNMTi_Sed", colnames(metabolomicsdata))

#Make a summarized experiment for just the treatment group of interest (not really
necessary here, but script is set up in case multiple groups have missing data)
```

```
sim_unique_names <- make_unique(metabolomicsdata, "name", "ID", delim = ";")
se <- make_se(sim_unique_names, c(NNMTi_Sed), experimental_design)

#Shows you how many metabolites are missing data
plot_frequency(se)

no_filter <- se

#Shows the pattern of how the metabolites are missing (can help one determine whether
metabolites are missing at random [MAR] or missing not at random [MNAR])
plot_missval(no_filter)

#To see a density and culumative summary plots of metabolites with and without missing
values (not relevant when only one metabolite is missing a value)
plot_detect(no_filter)


#Copied directly from:
https://www.bioconductor.org/packages/devel/bioc/vignettes/DEP/inst/doc/MissingValues.h
tml
#How the data looks without imputation
no_imputation <- no_filter

#Imputes the missing data via random draws from a Gaussian distribution centered around
a minimal value (this is ideal for data that's MNAR)
MinProb_imputation <- impute(no_filter, fun = "MinProb", q = 0.01)

#Imputes the missing data via random draws from a manually-defined left-shifted
Gaussian distribution (this is ideal for data that's MNAR)
manual_imputation <- impute(no_filter, fun = "man", shift = 1.8, scale = 0.3)

#Imputes the missing data via the k-nearest neighbor approach (this is ideal for data that's
MAR)
knn_imputation <- impute(no_filter, fun = "knn", rowmax = 0.9)

#Getting fancy fancy with it-- running a mixed imputation with the the appropriate
imputation methods for the MAR and MNAR data
#(as opposed to above, where all metabolites under MAR methods or all undergo MNAR
methods)
metabolites_MNAR <- get_df_long(no_filter) %>%
  group_by(name, condition) %>%
  summarize(NAs = all(is.na(intensity))) %>%
  filter(NAs) %>%
  pull(name) %>%
  unique()

MNAR <- names(no_filter) %in% metabolites_MNAR

mixed_imputation <- impute(
  no_filter,
  fun = "mixed",
```

```
  randna = !MNAR,
  mar = "knn",
  mnar = "zero")

#See how the density distributions differ before imputation compared to each of the various imputation methods
plot_imputation(no_filter, MinProb_imputation,
                manual_imputation, knn_imputation, mixed_imputation)

#Format the mixed imputation data and export it to a csv file
mixed_imputation_export <- assay(mixed_imputation)
write.csv(mixed_imputation_export, "[file name .csv complete with path to desired file location]", row.names = TRUE)
```

R script to perform Metabolomics_Step2A_SL+TMMNormalization&DEanalysis_PositiveIonMode

```
#Script taken from: https://pwilmart.github.io/IRS_normalization/understanding_IRS.html and modified according to the needs of this respective project
#All credit for building out the IRS platform, and the serial steps to normalize the data goes to Phillip Wilmarth

#Load the libraries
library(tidyverse)
library(limma)
library(edgeR)
library(sva)
library(readr)
library(stringr)
library(psych)


rawdata <- read_tsv("[path to file]")


#Save the metabolite names but remove them from the data frame
annotate_df <- rawdata[1]
data_raw <- as.data.frame(rawdata[2:30])
row.names(data_raw) <- annotate_df$`MetaboliteName`

#Create your groups and string them together
group <-
rep(c("NNMTi_Sed","Sed","Sed","Sed","NNMTi_Sed","NNMTi_Sed","Sed","Sed","NNMTi_Sed","NNMTi_Sed","Sed","Sed","NNMTi_Sed","Sed","NNMTi_Sed","NNMTi_Sed","PoWeR","NNMTi_PoWeR","NNMTi_PoWeR","PoWeR","NNMTi_PoWeR","PoWeR","PoWeR","NNMTi_PoWeR","NNMTi_PoWeR","NNMTi_PoWeR","NNMTi_PoWeR","PoWeR","PoWeR"))
group <- factor(group, levels = c("NNMTi_Sed","Sed","NNMTi_PoWeR", "PoWeR"))
str(group)

#Set the font size so it can fit all the info you'll want in the title
```

```
par(cex.main=0.8)

#Plot the raw data
boxplot(log2(data_raw), col = group, notch = TRUE, main = 'RAW DATA \nSed [black],
NNMTi_Sed [red], PoweR [green], NNMTi_PoWeR [blue]',
	xlab = 'Gastroc Samples', ylab = 'Log2 of Raw Data')


#First Normalization step: Sample Loading [SL] normalization
#Determine the global scaling value
target <- mean(c(colSums(data_raw)))
norm_facs <- target / colSums(data_raw)
data_sl <- sweep(data_raw, 2, norm_facs, FUN = "*")


#To visualize SL-normalized data
boxplot(log2(data_sl), col = group,
	notch = TRUE, main = "SAMPLE LOADING (SL) NORMALIZED DATA \n Sed [black],
NNMTi_Sed [red], PoWeR [green], NNMTi_PoWeR [blue]",
	xlab = 'Gastroc Samples', ylab = 'log2 of Raw SL-Normalized Peak Area')

plotDensities(log2(data_sl), group=group, col=c("black","red","green","blue"), main = "SL
Normalized Data")

#To see what's in the data frame or what the data visually looks like raw:
head(data_sl)

#Plot the data as a PCoA
col <- as.numeric(group)
plotMDS(log2(data_sl), labels=col,col=col,
	pch = 19, cex = 1, main = "SL-Normalized Data Clustering")

#To Label the PCoA plot a little bit better
col <- as.numeric(group)
plotMDS(log2(data_sl), col=col,
	pch = 19, cex = 1, main = "SL-Normalized Data Clustering")

#To see how similar the column (sample) totals are:
#format(round(colSums(data_raw), digits = 0), big.mark = ",")


#Second normalization step: TMM on the SL-normalized data
sl_tmm <- calcNormFactors(data_sl)
data_sl_tmm <- sweep(data_sl, 2, sl_tmm, FUN = "/")

boxplot(log2(data_sl_tmm), col = group,
	notch= TRUE, main = "SL+TMM NORMALIZED DATA \n Sed [black], NNMTi_Sed
[red], PoWer [green], NNMTi_PoWeR [blue]",
	xlab = 'Gastroc Samples', ylab = 'log2 of SL+TMM-normalized data')
```

```
plotDensities(log2(data_sl_tmm), group=group, col=c("black","red","green","blue"), main =
"SL+TMM Normalized Data")


#To see how similar the column (sample) totals are:
#format(round(colSums(data_sl_tmm), digits = 0), big.mark = ",")

#Generate a PCoA plot
col <- as.numeric(group)
plotMDS(log2(data_sl_tmm), labels=col,col=col,
	pch = 19, cex = 1, main = "SL+TMM Clustering")

#To Label the PCoA plot a little bit better
col <- as.numeric(group)
plotMDS(log2(data_sl_tmm), col=col,
	pch = 19, cex = 1, main = "SL+TMM Clustering")

#Save the results
write.csv(data_sl_tmm,"[file name .csv complete with path to desired file location]",
row.names = TRUE)

#print so you know the versions of all of the packages
sessionInfo()


#Generate your DGE list
y_SLtmm <- DGEList(counts = data_sl_tmm, group = group)

#Design a model matrix; since the group is a factor and not a covariate, the models with
and without the intercept are the same
(https://www.ncbi.nlm.nih.gov/pmc/articles/PMC7873980/)
study_design <- model.matrix(~0+group, data=y_SLtmm$samples)
colnames(study_design) <- levels(y_SLtmm$samples$group)
study_design

#Estimate dispersion (EdgeR tutorial)
y <- estimateDisp(y_SLtmm, study_design, robust=TRUE)
y$common.dispersion
plotBCV(y)

#Estimate QL dispersions
fit <- glmQLFit(y, study_design, robust=TRUE)

#Plot them if you want to
plotQLDisp(fit)

#Test for DE and correction for multiple comparisons: PoWeR (+/-NNMTi) vs. Sedentary
(+/-NNMTi)
qlf <- glmQLFTest(fit, contrast=c(-0.5,-0.5,0.5,0.5))
topTags(qlf)
FDR= p.adjust(qlf$table$PValue, method="BH")
```

```
results = data.frame(qlf, FDR)

#Save the results
write.csv(results,"[file name .csv complete with path to desired file location]", row.names =
TRUE)

#Test for DE and correction for multiple comparisons: NNMTi (+/-PoWeR) vs. Control (+/-
PoWeR)
qlf <- glmQLFTest(fit, contrast=c(0.5,-0.5,0.5,-0.5))
topTags(qlf)
FDR <- p.adjust(qlf$table$PValue, method="BH")
results = data.frame(qlf, FDR)

#Save the results
write.csv(results,"[file name .csv complete with path to desired file location]", row.names =
TRUE)

#Test for DE and correction for multiple comparisons: PoWeR NNMTi vs. PoWeR
qlf <- glmQLFTest(fit, contrast=c(0,0,1,-1))
topTags(qlf)
FDR <- p.adjust(qlf$table$PValue, method="BH")
results = data.frame(qlf, FDR)

#Save the results
write.csv(results,"[file name .csv complete with path to desired file location]", row.names =
TRUE)

#Test for DE and correct for multiple comparisons: Sedentary NNMTi vs. Sedentary
qlf <- glmQLFTest(fit, contrast=c(1,-1,0,0))
topTags(qlf)
FDR <- p.adjust(qlf$table$PValue, method="BH")
results = data.frame(qlf, FDR)

#Save the results
write.csv(results,"[file name .csv complete with path to desired file location]", row.names =
TRUE)

#Test for DE and correct for multiple comparisons: PoWeR vs. Sedentary
qlf <- glmQLFTest(fit, contrast=c(0,-1,0,1))
topTags(qlf)
FDR <- p.adjust(qlf$table$PValue, method="BH")
results = data.frame(qlf, FDR)

#Save the results
write.csv(results,"[file name .csv complete with path to desired file location]", row.names =
TRUE)

#Test for DE and correct for multiple comparisons: PoWeR NNMTi vs. Sedentary NNMTi
qlf <- glmQLFTest(fit, contrast=c(-1,0,1,0))
topTags(qlf)
FDR <- p.adjust(qlf$table$PValue, method="BH")
```

```
results = data.frame(qlf, FDR)

#Save the results
write.csv(results,"[file name .csv complete with path to desired file location]", row.names =
TRUE)

#Summarize your results at anytime with the formula below:
summary(decideTests(qlf))


#Test for DE and correct for multiple comparisons: PoWeR NNMTi vs. Sedentary Ctl
qlf <- glmQLFTest(fit, contrast=c(0,-1,1,0))
topTags(qlf)
FDR <- p.adjust(qlf$table$PValue, method="BH")
results = data.frame(qlf, FDR)

#Save the results
write.csv(results,"[file name .csv complete with path to desired file location]", row.names =
TRUE)

#Summarize your results at anytime with the formula below:
summary(decideTests(qlf))
```

R script to perform Metabolomics_Step2B_SL+TMMNormalization&DEanalysis_NegativeIonMode

```
#Script taken from: https://pwilmart.github.io/IRS_normalization/understanding_IRS.html
and modified according to the needs of this respective project
#All credit for building out the IRS platform, and the serial steps to normalize the data goes
to Phillip Wilmarth

#Load the libraries
library(tidyverse)
library(limma)
library(edgeR)
library(sva)
library(readr)
library(stringr)
library(psych)


rawdata <- read_tsv("[path to file]")


#Save the metabolite names but remove them from the data frame
annotate_df <- rawdata[1]
data_raw <- as.data.frame(rawdata[2:30])
row.names(data_raw) <- annotate_df$`MetaboliteName`
```

```
#Create your groups and string them together
group <-
rep(c("NNMTi_Sed","Sed","Sed","Sed","NNMTi_Sed","NNMTi_Sed","Sed","Sed","NNMTi_
Sed","NNMTi_Sed","Sed","Sed","NNMTi_Sed","Sed","NNMTi_Sed","NNMTi_Sed","PoWeR
","NNMTi_PoWeR","NNMTi_PoWeR","PoWeR","NNMTi_PoWeR","PoWeR","PoWeR","NN
MTi_PoWeR","NNMTi_PoWeR","NNMTi_PoWeR","NNMTi_PoWeR","PoWeR","PoWeR"))
group <- factor(group, levels = c("NNMTi_Sed","Sed","NNMTi_PoWeR", "PoWeR"))
str(group)

#Set the font size so it can fit all the info you'll want in the title
par(cex.main=0.8)

#Plot the raw data
boxplot(log2(data_raw), col = group, notch = TRUE, main = 'RAW DATA \nSed [black],
NNMTi_Sed [red], PoWeR [green], NNMTi_PoWeR [blue]',
	xlab = 'Gastroc Samples', ylab = 'Log2 of Raw Data')


#First Normalization step: Sample Loading [SL] normalization
#Determine the global scaling value
target <- mean(c(colSums(data_raw)))
norm_facs <- target / colSums(data_raw)
data_sl <- sweep(data_raw, 2, norm_facs, FUN = "*")


#To visualize SL-normalized data
boxplot(log2(data_sl), col = group,
	notch = TRUE, main = "SAMPLE LOADING (SL) NORMALIZED DATA \n Sed [black],
NNMTi_Sed [red], PoWeR [green], NNMTi_PoWeR [blue]",
	xlab = 'Gastroc Samples', ylab = 'log2 of Raw SL-Normalized Peak Area')

plotDensities(log2(data_sl), group=group, col=c("black","red","green","blue"), main = "SL
Normalized Data")

#To see what's in the data frame or what the data visually looks like raw:
head(data_sl)

#Plot the data as a PCoA
col <- as.numeric(group)
plotMDS(log2(data_sl), labels=col,col=col,
	pch = 19, cex = 1, main = "SL-Normalized Data Clustering")

#To Label the PCoA plot a little bit better
col <- as.numeric(group)
plotMDS(log2(data_sl), col=col,
	pch = 19, cex = 1, main = "SL-Normalized Data Clustering")

#To see how similar the column (sample) totals are:
#format(round(colSums(data_raw), digits = 0), big.mark = ",")
```

```
#Second normalization step: TMM on the SL-normalized data
sl_tmm <- calcNormFactors(data_sl)
data_sl_tmm <- sweep(data_sl, 2, sl_tmm, FUN = "/")

boxplot(log2(data_sl_tmm), col = group,
	notch= TRUE, main = "SL+TMM NORMALIZED DATA \n Sed [black], NNMTi_Sed
[red], PoWeR [green], NNMTi_PoWeR [blue]",
	xlab = 'Gastroc Samples', ylab = 'log2 of SL+TMM-normalized data')

plotDensities(log2(data_sl_tmm), group=group, col=c("black","red","green","blue"), main =
"SL+TMM Normalized Data")


#To see how similar the column (sample) totals are:
#format(round(colSums(data_sl_tmm), digits = 0), big.mark = ",")

#Generate a PCoA plot
col <- as.numeric(group)
plotMDS(log2(data_sl_tmm), labels=col,col=col,
	pch = 19, cex = 1, main = "SL+TMM Clustering")

#To Label the PCoA plot a little bit better
col <- as.numeric(group)
plotMDS(log2(data_sl_tmm), col=col,
	pch = 19, cex = 1, main = "SL+TMM Clustering")

#Save the results
write.csv(data_sl_tmm,"[file name .csv complete with path to desired file location]",
row.names = TRUE)

#print so you know the versions of all of the packages
sessionInfo()


#Generate your DGE list
y_SLtmm <- DGEList(counts = data_sl_tmm, group = group)

#Design a model matrix; since the group is a factor and not a covariate, the models with
and without the intercept are the same
(https://www.ncbi.nlm.nih.gov/pmc/articles/PMC7873980/)
study_design <- model.matrix(~0+group, data=y_SLtmm$samples)
colnames(study_design) <- levels(y_SLtmm$samples$group)
study_design

#Estimate dispersion (EdgeR tutorial)
y <- estimateDisp(y_SLtmm, study_design, robust=TRUE)
y$common.dispersion
plotBCV(y)

#Estimate QL dispersions
fit <- glmQLFit(y, study_design, robust=TRUE)
```

```
#Plot them if you want to
plotQLDisp(fit)

#Test for DE and correction for multiple comparisons: PoWeR (+/-NNMTi) vs. Sedentary
(+/-NNMTi)
qlf <- glmQLFTest(fit, contrast=c(-0.5,-0.5,0.5,0.5))
topTags(qlf)
FDR= p.adjust(qlf$table$PValue, method="BH")
results = data.frame(qlf, FDR)

#Save the results
write.csv(results,"[file name .csv complete with path to desired file location]", row.names =
TRUE)

#Test for DE and correction for multiple comparisons: NNMTi (+/-PoWeR) vs. Control (+/-
PoWeR)
qlf <- glmQLFTest(fit, contrast=c(0.5,-0.5,0.5,-0.5))
topTags(qlf)
FDR <- p.adjust(qlf$table$PValue, method="BH")
results = data.frame(qlf, FDR)

#Save the results
write.csv(results,"[file name .csv complete with path to desired file location]", row.names =
TRUE)

#Test for DE and correction for multiple comparisons: PoWeR NNMTi vs. PoWeR
qlf <- glmQLFTest(fit, contrast=c(0,0,1,-1))
topTags(qlf)
FDR <- p.adjust(qlf$table$PValue, method="BH")
results = data.frame(qlf, FDR)

#Save the results
write.csv(results,"[file name .csv complete with path to desired file location]", row.names =
TRUE)

#Test for DE and correct for multiple comparisons: Sedentary NNMTi vs. Sedentary
qlf <- glmQLFTest(fit, contrast=c(1,-1,0,0))
topTags(qlf)
FDR <- p.adjust(qlf$table$PValue, method="BH")
results = data.frame(qlf, FDR)

#Save the results
write.csv(results,"[file name .csv complete with path to desired file location]", row.names =
TRUE)

#Test for DE and correct for multiple comparisons: PoWeR vs. Sedentary
qlf <- glmQLFTest(fit, contrast=c(0,-1,0,1))
topTags(qlf)
FDR <- p.adjust(qlf$table$PValue, method="BH")
results = data.frame(qlf, FDR)
```

```
#Save the results
write.csv(results,"[file name .csv complete with path to desired file location]", row.names = TRUE)

#Test for DE and correct for multiple comparisons: PoWeR NNMTi vs. Sedentary NNMTi
qlf <- glmQLFTest(fit, contrast=c(-1,0,1,0))
topTags(qlf)
FDR <- p.adjust(qlf$table$PValue, method="BH")
results = data.frame(qlf, FDR)

#Save the results
write.csv(results,"[file name .csv complete with path to desired file location]", row.names = TRUE)

#Summarize your results at anytime with the formula below:
summary(decideTests(qlf))

#Test for DE and correct for multiple comparisons: PoWeR NNMTi vs. Sedentary
qlf <- glmQLFTest(fit, contrast=c(0,-1,1,0))
topTags(qlf)
FDR <- p.adjust(qlf$table$PValue, method="BH")
results = data.frame(qlf, FDR)

#Save the results
write.csv(results,"[file name .csv complete with path to desired file location]", row.names = TRUE)

#Summarize your results at anytime with the formula below:
summary(decideTests(qlf))
```

R script to perform Proteomics_Step1_Group-ComputedMixedImputations

```
install.packages("reader")
install.packages("DEP")
install.packages("dplyr")
install.packages("tidyr")
install.packages("purrr")
install.packages("ggplot2")
install.packages("SummarizedExperiment")

library("readr")
library("DEP")
library("tidyr")
library("purrr")
library("ggplot2")
library("SummarizedExperiment")
library("dplyr")
```

```
###########################Sed###########################
MS3data <- read_tsv("[path to file]")

# get column numbers
RepIntensity_columns <- grep("RepIntensity.", colnames(MS3data))


experimental_design <- data_frame(
  label = colnames(MS3data)[!colnames(MS3data) %in% c("name", "ID")],
  condition = c(
    rep("Sed", 8)),

  replicate = rep(1:8))

data_se <- make_se(MS3data, RepIntensity_columns, experimental_design)

Sed <- grep("Sed", colnames(MS3data))

sim_unique_names <- make_unique(MS3data, "name", "ID", delim = ";")
se <- make_se(sim_unique_names, c(Sed), experimental_design)

plot_frequency(se)

no_filter <- se

plot_missval(no_filter)

plot_detect(no_filter)


#Copied directly from:
https://www.bioconductor.org/packages/devel/bioc/vignettes/DEP/inst/doc/MissingValues.h
tml
# No imputation
no_imputation <- no_filter

# Impute missing data using random draws from a
# Gaussian distribution centered around a minimal value (for MNAR)
MinProb_imputation <- impute(no_filter, fun = "MinProb", q = 0.01)


# Impute missing data using random draws from a
# manually defined left-shifted Gaussian distribution (for MNAR)
manual_imputation <- impute(no_filter, fun = "man", shift = 1.8, scale = 0.3)

# Impute missing data using the k-nearest neighbour approach (for MAR)
knn_imputation <- impute(no_filter, fun = "knn", rowmax = 0.9)
#The effect of data imputation on the distributions can be visualized.

#Getting fancy
proteins_MNAR <- get_df_long(no_filter) %>%
```

```
  group_by(name, condition) %>%
  summarize(NAs = all(is.na(intensity))) %>%
  filter(NAs) %>%
  pull(name) %>%
  unique()

MNAR <- names(no_filter) %in% proteins_MNAR

mixed_imputation <- impute(
  no_filter,
  fun = "mixed",
  randna = !MNAR,
  mar = "knn",
  mnar = "zero")

# Plot intensity distributions before and after imputation
plot_imputation(no_filter, MinProb_imputation,
                manual_imputation, knn_imputation, mixed_imputation)

mixed_imputation_export <- assay(mixed_imputation)
write.csv(mixed_imputation_export, "[file name .csv complete with path to desired file
location]", row.names = TRUE)

##########################NNMTi_Sed##########################

MS3data <- read_tsv("[path to file]")

# get column numbers
RepIntensity_columns <- grep("RepIntensity.", colnames(MS3data))


experimental_design <- data_frame(
  label = colnames(MS3data)[!colnames(MS3data) %in% c("name", "ID")],
  condition = c(
    rep("NNMTi_Sed", 8)),

  replicate = rep(1:8))

data_se <- make_se(MS3data, RepIntensity_columns, experimental_design)

NNMTi_Sed <- grep("NNMTi_Sed", colnames(MS3data))

sim_unique_names <- make_unique(MS3data, "name", "ID", delim = ";")
se <- make_se(sim_unique_names, c(NNMTi_Sed), experimental_design)

plot_frequency(se)

no_filter <- se

plot_missval(no_filter)
```

```
plot_detect(no_filter)


#Copied directly from:
https://www.bioconductor.org/packages/devel/bioc/vignettes/DEP/inst/doc/MissingValues.h
tml
# No imputation
no_imputation <- no_filter

# Impute missing data using random draws from a
# Gaussian distribution centered around a minimal value (for MNAR)
MinProb_imputation <- impute(no_filter, fun = "MinProb", q = 0.01)


# Impute missing data using random draws from a
# manually defined left-shifted Gaussian distribution (for MNAR)
manual_imputation <- impute(no_filter, fun = "man", shift = 1.8, scale = 0.3)

# Impute missing data using the k-nearest neighbour approach (for MAR)
knn_imputation <- impute(no_filter, fun = "knn", rowmax = 0.9)
#The effect of data imputation on the distributions can be visualized.

#Getting fancy
proteins_MNAR <- get_df_long(no_filter) %>%
  group_by(name, condition) %>%
  summarize(NAs = all(is.na(intensity))) %>%
  filter(NAs) %>%
  pull(name) %>%
  unique()

MNAR <- names(no_filter) %in% proteins_MNAR

mixed_imputation <- impute(
  no_filter,
  fun = "mixed",
  randna = !MNAR,
  mar = "knn",
  mnar = "zero")

# Plot intensity distributions before and after imputation
plot_imputation(no_filter, MinProb_imputation,
                manual_imputation, knn_imputation, mixed_imputation)

mixed_imputation_export <- assay(mixed_imputation)
write.csv(mixed_imputation_export, "[file name .csv complete with path to desired file
location]", row.names = TRUE)

###########################PoWeR###########################
MS3data <- read_tsv("[path to file]")

# get column numbers
```

```
RepIntensity_columns <- grep("RepIntensity.", colnames(MS3data))


experimental_design <- data_frame(
  label = colnames(MS3data)[!colnames(MS3data) %in% c("name", "ID")],
  condition = c(
    rep("PoWeR", 7)),

  replicate = rep(1:7))

data_se <- make_se(MS3data, RepIntensity_columns, experimental_design)

PoWeR <- grep("PoWeR", colnames(MS3data))

sim_unique_names <- make_unique(MS3data, "name", "ID", delim = ";")
se <- make_se(sim_unique_names, c(PoWeR), experimental_design)

plot_frequency(se)

no_filter <- se

plot_missval(no_filter)

plot_detect(no_filter)


#Copied directly from:
https://www.bioconductor.org/packages/devel/bioc/vignettes/DEP/inst/doc/MissingValues.h
tml
# No imputation
no_imputation <- no_filter

# Impute missing data using random draws from a
# Gaussian distribution centered around a minimal value (for MNAR)
MinProb_imputation <- impute(no_filter, fun = "MinProb", q = 0.01)


# Impute missing data using random draws from a
# manually defined left-shifted Gaussian distribution (for MNAR)
manual_imputation <- impute(no_filter, fun = "man", shift = 1.8, scale = 0.3)

# Impute missing data using the k-nearest neighbour approach (for MAR)
knn_imputation <- impute(no_filter, fun = "knn", rowmax = 0.9)
#The effect of data imputation on the distributions can be visualized.

#Getting fancy
proteins_MNAR <- get_df_long(no_filter) %>%
  group_by(name, condition) %>%
  summarize(NAs = all(is.na(intensity))) %>%
  filter(NAs) %>%
  pull(name) %>%
```

```
  unique()

MNAR <- names(no_filter) %in% proteins_MNAR

mixed_imputation <- impute(
  no_filter,
  fun = "mixed",
  randna = !MNAR,
  mar = "knn",
  mnar = "zero")

# Plot intensity distributions before and after imputation
plot_imputation(no_filter, MinProb_imputation,
                manual_imputation, knn_imputation, mixed_imputation)

mixed_imputation_export <- assay(mixed_imputation)
write.csv(mixed_imputation_export, "[file name .csv complete with path to desired file
location]", row.names = TRUE)

##########################NNMTi_PoWeR##########################

MS3data <- read_tsv("[path to file]")

# get column numbers
RepIntensity_columns <- grep("RepIntensity.", colnames(MS3data))


experimental_design <- data_frame(
  label = colnames(MS3data)[!colnames(MS3data) %in% c("name", "ID")],
  condition = c(
    rep("NNMTi_PoWeR", 7)),

  replicate = rep(1:7))

data_se <- make_se(MS3data, RepIntensity_columns, experimental_design)

NNMTi_PoWeR <- grep("NNMTi_PoWeR", colnames(MS3data))

sim_unique_names <- make_unique(MS3data, "name", "ID", delim = ";")
se <- make_se(sim_unique_names, c(NNMTi_PoWeR), experimental_design)

plot_frequency(se)

no_filter <- se

plot_missval(no_filter)

plot_detect(no_filter)
```

```
#Copied directly from:
https://www.bioconductor.org/packages/devel/bioc/vignettes/DEP/inst/doc/MissingValues.h
tml
# No imputation
no_imputation <- no_filter

# Impute missing data using random draws from a
# Gaussian distribution centered around a minimal value (for MNAR)
MinProb_imputation <- impute(no_filter, fun = "MinProb", q = 0.01)


# Impute missing data using random draws from a
# manually defined left-shifted Gaussian distribution (for MNAR)
manual_imputation <- impute(no_filter, fun = "man", shift = 1.8, scale = 0.3)

# Impute missing data using the k-nearest neighbour approach (for MAR)
knn_imputation <- impute(no_filter, fun = "knn", rowmax = 0.9)
#The effect of data imputation on the distributions can be visualized.

#Getting fancy
proteins_MNAR <- get_df_long(no_filter) %>%
  group_by(name, condition) %>%
  summarize(NAs = all(is.na(intensity))) %>%
  filter(NAs) %>%
  pull(name) %>%
  unique()

MNAR <- names(no_filter) %in% proteins_MNAR

mixed_imputation <- impute(
  no_filter,
  fun = "mixed",
  randna = !MNAR,
  mar = "knn",
  mnar = "zero")

# Plot intensity distributions before and after imputation
plot_imputation(no_filter, MinProb_imputation,
                manual_imputation, knn_imputation, mixed_imputation)

mixed_imputation_export <- assay(mixed_imputation)
write.csv(mixed_imputation_export, "[file name .csv complete with path to desired file
location]", row.names = TRUE)
```

R script to perform
Proteomics_Step2_Normalization_ReporterIntensity_SL+TMM+IRS

```
#Script taken from: https://pwilmart.github.io/IRS_normalization/understanding_IRS.html
and modified according to the needs of this respective project
#All credit for building out the IRS platform, and the serial steps to normalize the data goes
to Phillip Wilmarth

#Load the libraries
library(tidyverse)
library(limma)
library(edgeR)
library(sva)
library(readr)
library(stringr)
library(psych)


MS3data <- read_tsv("[path to file]")


#Save the protein accession annotations but remove them from the data frame
annotate_df <- MS3data[1]
data_raw <- as.data.frame(MS3data[2:33])
row.names(data_raw) <- annotate_df$`Protein Accession No.`

#Create your groups and string them together
group <- rep(c("NNMTi_Sed", "Sed", "Sed", "Sed", "NNMTi_Sed", "NNMTi_Sed", "Sed",
"Sed", "NNMTi_Sed", "NNMTi_Sed", "Sed", "Sed", "NNMTi_Sed", "Sed", "NNMTi_Sed",
"NNMTi_Sed", "PoWeR", "NNMTi_PoWeR", "NNMTi_PoWeR", "PoWeR",
"NNMTi_PoWeR", "PoWeR", "PoWeR", "NNMTi_PoWeR", "NNMTi_PoWeR",
"NNMTi_PoWeR", "NNMTi_PoWeR", "PoWeR", "PoWeR",
"PoWeR","Pool_Set1","Pool_Set2"))
group <- factor(group, levels = c("Sed","NNMTi_Sed","PoWeR",
"NNMTi_PoWeR","Pool_Set1","Pool_Set2"))
str(group)

#Set the font size so it can fit all the info you'll want in the title
par(cex.main=0.8)

#Plot the raw data
boxplot(log2(data_raw), col = group, notch = TRUE, main = 'RAW DATA \nBatch1 (Sed
[black], NNMTi_Sed [red]), \nBatch2 (PoWeR [green], NNMTi_PoWeR [blue]), \nPooled
(Batch1 [cyan], Batch2 [purple])',
        xlab = 'Gastroc Samples', ylab = 'log2 of Intensity')

#To see what's in the data frame or what the data visually looks like raw:
head(data_raw)

#Plot the data as a PCoA
col <- as.numeric(group)
plotMDS(log2(data_raw), labels=col,col=col,
        pch = 19, cex = 1, main = "Raw Data Clustering")
```

```
#To Label the PCoA plot a little bit better
col <- as.numeric(group)
plotMDS(log2(data_raw), col=col,
	pch = 19, cex = 1, main = "Raw Data Clustering")

#To see how similar the column (sample) totals are:
#format(round(colSums(data_raw), digits = 0), big.mark = ",")

#Split the TMT data by batch
batch1_raw <- data_raw[c(1:16,31)]
batch2_raw <- data_raw[c(17:30,32)]

#First Normalization step: adjust each TMT experiment to equal signal per channel
(Sample Loading [SL] normalization)
#Determine the global scaling value
target <- mean(c(colSums(batch1_raw[1:16]), colSums(batch2_raw[1:14])))
norm_facs <- target / colSums(batch1_raw)
batch1_sl <- sweep(batch1_raw, 2, norm_facs, FUN = "*")
norm_facs <- target / colSums(batch2_raw)
batch2_sl <- sweep(batch2_raw, 2, norm_facs, FUN = "*")

#Aggregate it
data_sl <- cbind(batch1_sl, batch2_sl)

#Drop the pooled average samples to decide the reference column for
Tdata_sl_sanspooled <- c(data_sl[1:16], data_sl[18:31])
data_sl_sanspooled <- data_sl[c(1:16,18:31)]
refColumn <- calcNormFactors(data_sl_sanspooled)
refColumn
#Iterate through 1:30 until the values match refColumn
isitthisone <- calcNormFactors(data_sl_sanspooled, refColumn=1)
isitthisone

#In our case, it was Sample 23, PoWeR_24_Set2; you'll use this later to set the reference
column for TMM
#This obnoxious step was used to avoid using an average to calculate the TMM; if you're
comfortable using
#one of the pooled averages as a reference value for TMM, we found that in our dataset
sample 17, or Pooled_Set1,
#was the most representative of our data. We chose not use this, since it is not necessarily
most representative
#the mean, since it is a mean of other samples

#Now that you've aggregated it differently, your Pooled_set1 has shifted from 31 to 17. You
need to re-group!
group2 <- rep(c("NNMTi_Sed", "Sed", "Sed", "Sed", "NNMTi_Sed", "NNMTi_Sed", "Sed",
"Sed", "NNMTi_Sed", "NNMTi_Sed", "Sed", "Sed", "NNMTi_Sed", "Sed", "NNMTi_Sed",
"NNMTi_Sed","Pool_Set1", "PoWeR", "NNMTi_PoWeR", "NNMTi_PoWeR", "PoWeR",
"NNMTi_PoWeR", "PoWeR", "PoWeR", "NNMTi_PoWeR", "NNMTi_PoWeR",
"NNMTi_PoWeR", "NNMTi_PoWeR", "PoWeR", "PoWeR", "PoWeR","Pool_Set2"))
```

```
group2 <- factor(group2, levels = c("Sed","NNMTi_Sed","PoWeR",
"NNMTi_PoWeR","Pool_Set1","Pool_Set2"))
str(group2)

#To visualize SL-normalized data
boxplot(log2(data_sl), col = group2,
	notch = TRUE, main = "SAMPLE LOADING (SL) NORMALIZED DATA \nBatch1 (Sed
[black], NNMTi_Sed [red]), \nBatch2 (PoWeR [green], NNMTi_PoWeR [blue]), \nPooled
(Batch1 [cyan], Batch2 [purple])",
	xlab = 'Gastroc Samples', ylab = 'log2 of Intensity')

plotDensities(log2(data_sl), group=group2,
col=c("black","red","green","blue","cyan","purple"), main = "SL Normalized Data")

#To see how similar the column (sample) totals are:
#format(round(colSums(data_sl), digits = 0), big.mark = ",")

#Second normalization step: TMM on the SL-normalized data
#Because the pooled samples are retained in data_sl relative to data_sl_sanspooled, the
relevant refColumn is actually 24
sl_tmm <- calcNormFactors(data_sl, refColumn=24)
data_sl_tmm <- sweep(data_sl, 2, sl_tmm, FUN = "/")

boxplot(log2(data_sl_tmm), col = group2,
	notch= TRUE, main = "TMM+SL NORMALIZED DATA \nBatch1 (Sed [black],
NNMTi_Sed [red]), \nBatch2 (PoWeR [green], NNMTi_PoWeR [blue]), \nPooled (Batch1
[cyan], Batch2 [purple])",
	xlab = 'Gastroc Samples', ylab = 'log2 of Intensity')

plotDensities(log2(data_sl_tmm), group=group2,
col=c("black","red","green","blue","cyan","purple"), main = "TMM+SL Normalized Data")


#To see how similar the column (sample) totals are:
#format(round(colSums(data_sl_tmm), digits = 0), big.mark = ",")

#Generate a PCoA plot
col <- as.numeric(group2)
plotMDS(log2(data_sl_tmm), labels=col,col=col,
	pch = 19, cex = 1, main = "SL+TMM Clustering")

#To Label the PCoA plot a little bit better
col <- as.numeric(group2)
plotMDS(log2(data_sl_tmm), col=col,
	pch = 19, cex = 1, main = "SL+TMM Clustering Reporter Intensity Data")

#Third normalization step: internal reference scaling (IRS) on the SL-TMM normalized data
#Identify the columns of data which are serving as the internal references for each batch
(the averages of samples #4 and #10) then perform the IRS itself
irs_factors <- data.frame(PoolBatch1 = rowMeans(data_sl_tmm[17]), PoolBatch2 =
rowMeans(data_sl_tmm[32]))
```

```
irs_factors$geomean <- apply(irs_factors, 1, function(x) exp(mean(log(x))))
irs_factors$fac1 <- irs_factors$geomean / irs_factors$PoolBatch1
irs_factors$fac2 <- irs_factors$geomean / irs_factors$PoolBatch2

#To see what's in the data frame or what the data visually looks like at present:
head(irs_factors)

all_irs <- data_sl_tmm[1:16] * irs_factors$fac1
all_irs <- cbind(all_irs, data_sl_tmm[18:31] * irs_factors$fac2)

#Again, you've messed up your groups when you aggregated. Re-group!
group3 <- rep(c("NNMTi_Sed", "Sed", "Sed", "Sed", "NNMTi_Sed", "NNMTi_Sed", "Sed",
"Sed", "NNMTi_Sed", "NNMTi_Sed", "Sed", "Sed", "NNMTi_Sed", "Sed", "NNMTi_Sed",
"NNMTi_Sed","PoWeR", "NNMTi_PoWeR", "NNMTi_PoWeR", "PoWeR",
"NNMTi_PoWeR", "PoWeR", "PoWeR", "NNMTi_PoWeR", "NNMTi_PoWeR",
"NNMTi_PoWeR", "NNMTi_PoWeR", "PoWeR", "PoWeR", "PoWeR"))
group3 <- factor(group3, levels = c("Sed","NNMTi_Sed","PoWeR", "NNMTi_PoWeR"))
str(group3)

#Visualize the SL-TMM-IRS-normalized data
boxplot(log2(all_irs), col = group3,
        notch= TRUE, main = "TMM+SL+IRS NORMALIZED REPORTER INTENSITY DATA
\nBatch1 (Sed [black], NNMTi_Sed [red]), \nBatch2 (PoWeR [green], NNMTi_PoWeR
[blue])",
        xlab = 'Gastroc Samples', ylab = 'log2 of Intensity')

plotDensities(log2(all_irs), group=group3, col=c("black","red","green","blue"), main =
"TMM+SL+IRS Normalized Reporter Intensity Data")


#Plot the data as a PCoA
#col2 <- as.numeric(group3)
#plotMDS(log2(all_irs), labels=col2,col=col2,
#        pch = 19, cex = 1, main = "SL+TMM+IRS Clustering")

#To Label the PCoA plot a little bit better
col2 <- as.numeric(group3)
plotMDS(log2(all_irs), col=col2,
        pch = 19, cex = 1, main = "SL+TMM+IRS Reporter Intensity Data Clustering")

#Save the results
write.csv(all_irs,"[file name .csv complete with path to desired file location]", row.names =
TRUE)

#print so you know the versions of all of the packages
sessionInfo()

#String your groups together
group4 <- factor(group3, levels = c("Sed","NNMTi_Sed","PoWeR", "NNMTi_PoWeR"))
str(group4)
```

```
#Generate your DGE list
y_irs <- DGEList(counts = all_irs, group = group4)

#Design a model matrix; since the group is a factor and not a covariate, the models with
and without the intercept are the same
(https://www.ncbi.nlm.nih.gov/pmc/articles/PMC7873980/)
study_design <- model.matrix(~0+group, data=y_irs$samples)
colnames(study_design) <- levels(y_irs$samples$group)
study_design

#Estimate dispersion (EdgeR tutorial)
y <- estimateDisp(y_irs, study_design, robust=TRUE)
y$common.dispersion
plotBCV(y)

#Estimate QL dispersions
fit <- glmQLFit(y, study_design, robust=TRUE)

#Plot them if you want to
plotQLDisp(fit)

#Test for DE and correction for multiple comparisons: PoWeR (+/-NNMTi) vs. Sedentary
(+/-NNMTi)
qlf <- glmQLFTest(fit, contrast=c(-0.5,-0.5,0.5,0.5))
topTags(qlf)
FDR= p.adjust(qlf$table$PValue, method="BH")
results = data.frame(qlf, FDR)

#Save the results
write.csv(results,"[file name .csv complete with path to desired file location]", row.names =
TRUE)

#Test for DE and correction for multiple comparisons: NNMTi (+/-PoWeR) vs. Control (+/-
PoWeR)
qlf <- glmQLFTest(fit, contrast=c(-0.5,0.5,-0.5,0.5))
topTags(qlf)
FDR <- p.adjust(qlf$table$PValue, method="BH")
results = data.frame(qlf, FDR)

#Save the results
write.csv(results,"[file name .csv complete with path to desired file location]", row.names =
TRUE)

#Test for DE and correction for multiple comparisons: PoWeR NNMTi vs. PoWeR
qlf <- glmQLFTest(fit, contrast=c(0,0,-1,1))
topTags(qlf)
FDR <- p.adjust(qlf$table$PValue, method="BH")
results = data.frame(qlf, FDR)

#Save the results
```

```
write.csv(results,"[file name .csv complete with path to desired file location]", row.names =
TRUE)

#Test for DE and correct for multiple comparisons: Sedentary NNMTi vs. Sedentary
qlf <- glmQLFTest(fit, contrast=c(-1,1,0,0))
topTags(qlf)
FDR <- p.adjust(qlf$table$PValue, method="BH")
results = data.frame(qlf, FDR)

#Save the results
write.csv(results,"[file name .csv complete with path to desired file location]", row.names =
TRUE)

#Test for DE and correct for multiple comparisons: PoWeR vs. Sedentary
qlf <- glmQLFTest(fit, contrast=c(-1,0,1,0))
topTags(qlf)
FDR <- p.adjust(qlf$table$PValue, method="BH")
results = data.frame(qlf, FDR)

#Save the results
write.csv(results,"[file name .csv complete with path to desired file location]", row.names =
TRUE)

#Test for DE and correct for multiple comparisons: PoWeR NNMTi vs. Sedentary NNMTi
qlf <- glmQLFTest(fit, contrast=c(0,-1,0,1))
topTags(qlf)
FDR <- p.adjust(qlf$table$PValue, method="BH")
results = data.frame(qlf, FDR)

#Save the results
write.csv(results,"[file name .csv complete with path to desired file location]", row.names =
TRUE)

#Summarize your results at anytime with the formula below:
summary(decideTests(qlf))

qlf <- glmQLFTest(fit, contrast=c(-1,0,0,1))
topTags(qlf)
FDR <- p.adjust(qlf$table$PValue, method="BH")
results = data.frame(qlf, FDR)

#Save the results
write.csv(results,"[file name .csv complete with path to desired file location]", row.names =
TRUE)
```

Matlab code to calculate Fatiguability (number of contractions above 50% and 70% peak torque)

```
%load data
Mouse_function_data = '..\Function data\*.txt'

%view directory
directory = dir(Mouse_function_data)

%storage for loop iterations

peak_torque1_vals = zeros(numel(directory),1) file_names =
cell(numel(directory),1)

%Looping through files

%number of files is assigned to directory for
file_counter = 1 : numel(directory)

    %setting folders and files to index file counter - pull data one at a
    %time

        folder_name = directory(file_counter).folder file_name
        = directory(file_counter).name full_name =
        fullfile(folder_name, file_name)

        %read table during each iteration t =
        readtable(full_name) justforce =
        t.FilteredForce

        %pull peak torque from each contraction peak_torque
        = max(justforce)

        %store peak torque from each contraction
        peak_torque1_vals(file_counter) = peak_torque

    end
```

```
%find max force for all contractions
peak_torque1 = max(peak_torque1_vals)


%find fifty percent of max force
fifty_percent_peak_torque = .5*peak_torque1
number_of_reps = 0


%second for loop to find 50% decrement
    for decrement_analyses = 1:numel(peak_torque1_vals)
        looping_vector = peak_torque1_vals(decrement_analyses)

        if looping_vector >= fifty_percent_peak_torque
             number_of_reps = number_of_reps + 1
        else
        end
    end


number_of_reps
```

Cumulative work code

```
%load data
Mouse_function_data_ = '..\Function data\*.txt'


%view directory
directory = dir(Mouse_function_data_)
total_force_vals = zeros(numel(directory),1)
peak_torque_vals = zeros(numel(directory),1)
file_names = cell(numel(directory),1)


for file_counter = 1:numel(directory)


    %set indices as files and folders in directory
    folder_name = directory(file_counter).folder
    file_name = directory(file_counter).name
    full_name = fullfile(folder_name,file_name)

    %pulling table
    t = readtable(full_name)
```

```
%pulling force
justforce = t.FilteredForce


%pull first 2000 elements of force vector - for average "resting force" justforce_baseline =
justforce(1:2000)
justforceavg = mean(justforce_baseline)


%pull peak torque too peak_torque =
max(justforce)


%assign time vector - total time is .75s here, not .7s like plantar
%flexion, hence different time vector sampling_rate =
750/7500
time_vector = [0:sampling_rate:749.99]'


%combine force and time vectors force_and_time =
[justforce,time_vector]
%7500 samples instead of 7000


%time index 1 for integration = 249.99, time index 2 = 599.99
%integral
lims1 = (time_vector>=249.99) & (time_vector<=599.99)
Total_work = trapz(time_vector(lims1),justforce(lims1))

%making theoretical "box" for correction factor - average resting work
%times the amount of time that we're integrating for force (350ms) Total_corrected_work =
Total_work - justforceavg*350


%store total work in empty vector total_force_vals(file_counter) =
Total_corrected_work peak_torque_vals(file_counter) =
peak_torque file_names{file_counter} = file_name


end


a = table(file_names,total_force_vals,peak_torque_vals) disp(a)

sum(total_force_vals)
max(peak_torque_vals)
```

Code for representative graphs

```
%load data for Nip11
Mouse_function_data = '..\Nip11 Function data\*.txt'


%view power directory
Nip11_directory = dir(Mouse_function_data)


%set 0 vectors to store peak torque for contractions
%power vectors
Nip11file_names = cell(numel(Nip11_directory),1)
Nip11peak_torque_vals = zeros(numel(Nip11_directory),1)


%loop through files in directory
for file_counter_1 = 1:numel(Nip11_directory)


    %setting folders and files to index file counter - pull data one at a
    %time
        folder_name1 = Nip11_directory(file_counter_1).folder
        file_name1 = Nip11_directory(file_counter_1).name
        full_name1 = fullfile(folder_name1, file_name1)


        %read table during each iteration t =
        readtable(full_name1) justforce1 =
        t.FilteredForce


        %pull peak torque from each contraction peak_torque1
        = max(justforce1)


        %store peak torque from each contraction Nip11peak_torque_vals(file_counter_1) =
        peak_torque1 Nip11file_names{file_counter_1} = file_name1


end





Mouse_function_data2 = '..\Nips2L Function data\*.txt'
```

```
%view sed directory
Nips2L_directory = dir(Mouse_function_data2)


%sed vectors
Nips2Lfile_names = cell(numel(Nips2L_directory),1)
Nips2Lpeak_torque_vals = zeros(numel(Nips2L_directory),1)


%loop through files in directory
for file_counter_2 =  1:numel(Nips2L_directory)


    %setting folders and files to index file counter - pull data one at a
    %time
        folder_name2 = Nips2L_directory(file_counter_2).folder
        file_name2 = Nips2L_directory(file_counter_2).name
        full_name2 = fullfile(folder_name2, file_name2)


        %read table during each iteration t =
        readtable(full_name2) justforce2 =
        t.FilteredForce


        %pull peak torque from each contraction peak_torque2
        = max(justforce2)


        %store peak torque from each contraction Nips2Lpeak_torque_vals(file_counter_2) =
        peak_torque2 Nips2Lfile_names{file_counter_2} = file_name2


end


table(Nip11file_names,Nip11peak_torque_vals,Nips2Lfile_names,Nips2Lpeak_torque_va ls)

%making fatigue graph
Number_of_contractions = [1:1:54]'


x = Number_of_contractions y1
= Nip11peak_torque_vals y2 =
Nips2Lpeak_torque_vals


clf
```

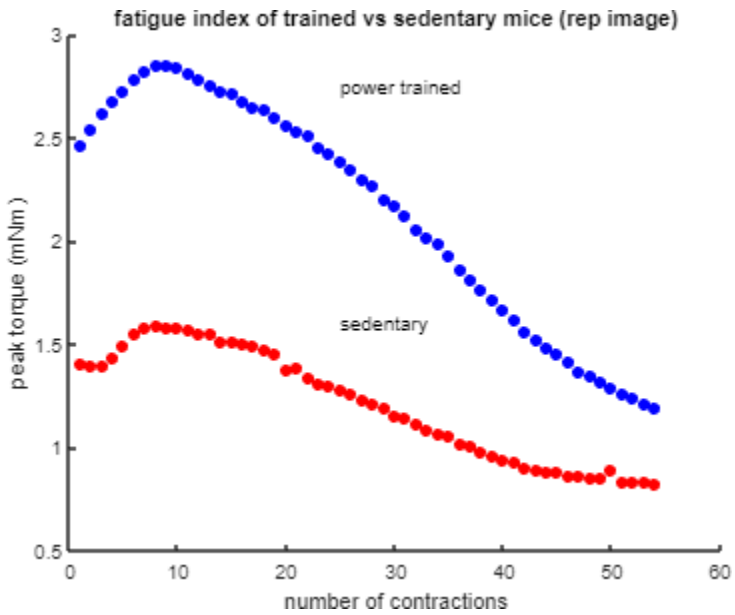


```
%Load directory
Nip11_data =  '..\Nip11 Function data\Nip11 Sumbax 08.txt'
Nips2L_data = '..\Nips2L Function data\Nips 2L submax 07.txt'


%read data
Nip11_vals = readtable(Nip11_data)
Nips2L_vals = readtable(Nips2L_data)


%pull force values
Nip11_force_vals = Nip11_vals.FilteredForce
Nips2L_force_vals = Nips2L_vals.FilteredForce


%Make time component
```

```
sampling_rate = 750/7500
time_vector = [0:sampling_rate:749.99]'


%making force trace graph
clf scatter(time_vector,Nip11_force_vals,'bo') hold on
scatter(time_vector,Nips2L_force_vals,'ro')




xlabel('time (ms)')
ylabel('peak torque (mNm)')
title('Representative force traces in trained vs sedentary mice') text(80,2,'blue =
trained')
text(80,1.85,'red = sedentary')
```

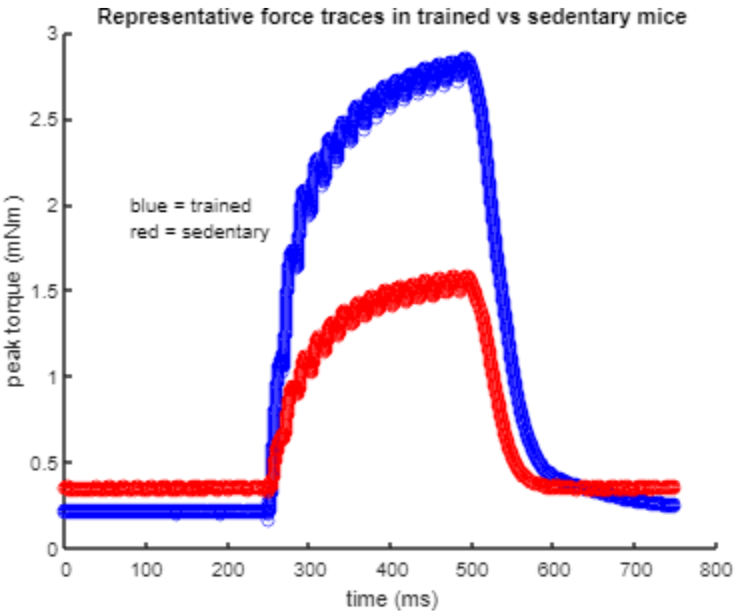

Supplement: Supplementary file 4 — Supplementary Information 2. [file 41598_2024_66034_MOESM4_ESM.pdf]
